# Engineered Endosymbionts that Modulate Primary Macrophage Function and Attenuate Tumor Growth by Shifting the Tumor Microenvironment

**DOI:** 10.1021/acsabm.5c00590

**Published:** 2025-06-24

**Authors:** Cody S. Madsen, Ashley V. Makela, Chima V. Maduka, Emily M. Greeson, Anthony Tundo, Evran Ural, Satyajit Hari Kulkarni, Ahmed A. Zarea, Matti Kiupel, Maryam Sayadi, Christopher H. Contag

**Affiliations:** 1 Nuclear and Chemical Sciences Division, Lawrence Livermore National Laboratory, 7000 East Avenue, Livermore, California 94550, United States; 2 Institute for Quantitative Health Science and Engineering, 3078 Michigan State University, 775 Woodlot Drive, East Lansing, Michigan 48823, United States; 3 Department of Biomedical Engineering, 3078 Michigan State University, 775 Woodlot Drive, East Lansing, Michigan 48823, United States; 4 Comparative Medicine & Integrative Biology, 3078 Michigan State University, 775 Woodlot Drive, East Lansing, Michigan 48823, United States; 5 Department of Microbiology, Genetics and Immunology, 3078 Michigan State University, 775 Woodlot Drive, East Lansing, Michigan 48823, United States; 6 Department of Biochemistry and Molecular Biology, 3078 Michigan State University, 775 Woodlot Drive, East Lansing, Michigan 48823, United States; 7 Program in Cellular and Molecular Biology, 3078 Michigan State University, 775 Woodlot Drive, East Lansing, Michigan 48823, United States; 8 Department of Pathobiology and Diagnostic Investigation, 3078 Michigan State University, 775 Woodlot Drive, East Lansing, Michigan 48823, United States

**Keywords:** engineered endosymbiont, transcription factors, bone marrow-derived macrophages, immune modulation, bacterial immunotherapy, tumor microenvironment, bacteriotherapy, immunometabolism

## Abstract

Modulating gene expression in macrophages can be used to improve tissue regeneration and redirect tumor microenvironments (TMEs) toward positive therapeutic outcomes. We have developed as an engineered endosymbiont (EES) capable of residing inside the eukaryotic host cell cytoplasm and controlling the fate of macrophages. Secretion of mammalian transcription factors (TFs) from that expresses listeriolysin O (LLO; allowing the EES to escape destruction by the macrophage) modulated expression of surface markers, cytokines, and chemokines, indicating functional changes in a macrophage/monocyte cell line. The engineered LLO TF strains were evaluated in murine bone marrow-derived macrophages (BMDMs) by flow cytometry, chemokine/cytokine profiling, metabolic assays, and RNA-Seq delivery of TFs by the EES shifted BMDM gene expression, production of cytokine and chemokines, and metabolic patterns, indicating that the TF strains could guide primary macrophage function. Thereafter, the ability of the TF strains to alter the TME was characterized in vivo in an orthotopic murine model of triple-negative breast cancer to assess therapeutic effects. The TF strains altered the TME by shifting immune cell composition and attenuating tumor growth. Additionally, multiple doses of the TF strains were well-tolerated by the mice. The use of LLO TF strains as EES showed promise as a unique cancer immunotherapy by directing the immune function intracellularly. The uses of EES could be expanded to modulate other mammalian cells over a range of biomedical applications.

## Graphical abstract

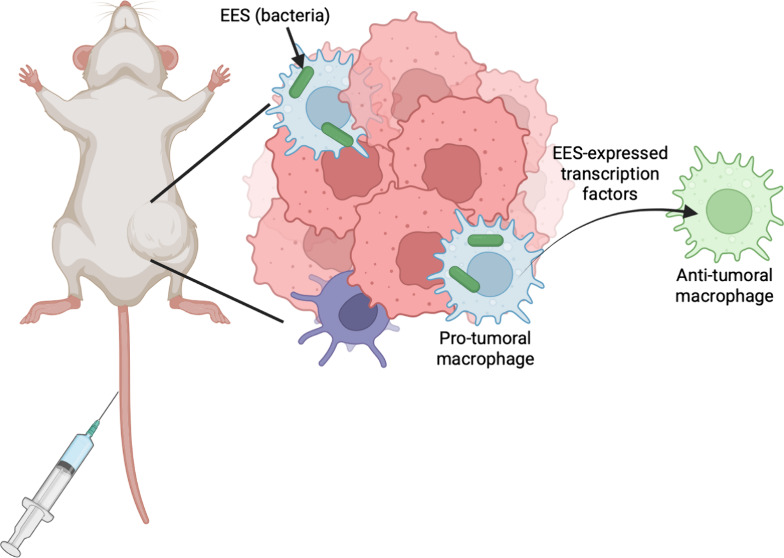

## Introduction

The concept of using endosymbionts and symbionts for modifying eukaryotic organisms to improve human health has been utilized in insects and nematodes^1^ *Wolbachia spp.* is a model endosymbiont that lives symbiotically within mosquitoes and naturally blocks the transmission of dengue and Zika virus by the mosquito species ,^2^ which has been used to stop transmission of these viruses in the environment.^2,3^ Various symbionts have been engineered for applications ranging from improving honeybee immunity^4^ to enhancing nematode biocontrol of dangerous crop pathogens.^5^ Furthermore, the benefits of symbiotic relationships between natural endosymbionts of invertebrates have been studied and have revealed clinically relevant compounds.^6,7^ Previously, we developed and tested nonpathogenic engineered endosymbionts (EES) from a chassis that expresses listeriolysin O (LLO) and can persist in the cytoplasm of mammalian cells.^8^ Variations of LLO expressing transcription factors (TFs) that altered macrophage function were delivered to a monocyte/macrophage cell line to study LLO-mediated entry into the host cell cytoplasm and modulation of macrophage pro- or anti-inflammatory responses.^8^

Bone marrow-derived macrophages (BMDMs)^9,10^ represent primary, antigen-presenting cells that signal to other important immune cells and regulate immune response^11,12^ and may better represent the in vivo response than immortalized cell lines. Macrophages, including BMDMs, can function across a spectrum from being pro-inflammatory (M1) to anti-inflammatory (M2).^13,14^ These shifts in function can be distinguished by changes in gene expression, cell surface markers, and expression of cytokines/chemokines.^15–18^ Furthermore, shifts in cellular metabolism underlie macrophage polarization and immune function, such as after activation by lipopolysaccharide (LPS) or other proinflammatory stimuli.^19–26^ Changes in cellular metabolism can be revealed by measuring markers of transition from oxidative phosphorylation to glycolysis including change in oxygen consumption rate (OCR), extracellular acidification rate (ECAR), and adenosine triphosphate (ATP) production rate.^27–30^ Metabolic shifts in macrophages occur as a component of the dynamic response to stimuli; thus, metabolic markers are indicative of macrophage responses. BMDMs have been used to study macrophage function in cancer,^31–33^ chronic inflammation,^34,35^ drug delivery,^36,37^ pathogen response,^38,39^ and tissue regeneration.^37,40^

Bacterial therapies based on extracellular bacteria have been developed to improve human health from improving the gut microbiome^41,42^ to treating cancer.^43–46^ (Bacille Calmette-Guerin, BCG)^47,48^ was originally developed as a tuberculosis vaccine but has now been approved for bladder cancer treatment, and other oncology applications are being tested.^43–46,49–51^ Furthermore, several advancements have been made in improving extracellular bacteria treatment of cancer from tropism to therapeutic delivery.^52–56^ Nissle 1917 (EcN) is a probiotic Gram-negative bacterium that has been part of the advancements in extracellular bacterial cancer immunotherapy.^57–61^ EcN has been modified to deliver chemotherapeutic drugs and proteins while improving safety as a probiotic.^56–60^ Intracellular bacteria have also been used in cancer immunotherapy. Gram-positive intracellular bacterium, , has been used to mobilize the immune system to alter the cancer microenvironment, and has been used extensively to disrupt viability of cancer cells along with therapeutic molecule delivery.^62–68^ Yet, these therapies are limited by acquired tolerance to live bacteria, especially Gram-negative bacteria, use of known pathogens as chassis organisms, and lack of characterized mechanisms on the target microenvironment.^69–71^ LLO,^72,73^ chassis of the EES, is derived from a nonpathogenic, generally recognized as safe (GRAS), Gram-positive, soil bacterium that respires as a facultative anaerobe and does not have a lipopolysaccharide (LPS)-mediated immune response, which provides an alternative to some of the challenges described above.^74,75^ The EES concept of using an intracellular approach to stimulate macrophages and mobilize the immune system to alter the cancer microenvironment parallels some efforts using^62,63^ for cancer treatment but is built on a nonpathogenic bacterial platform that has been classically used for secretion of complex proteins,^76^ and uniquely, it has been tied into mammalian cell regulatory pathways to redirect cell fates.^8^

In the therapies mentioned above, bacteria have been used to deliver a variety of therapeutic molecules including checkpoint inhibitors, nanobodies, or epitopes for vaccines and have even been used to cause tumor cell lysis as a way of altering the tumor microenvironment (TME) or to activate immune cells.^52–54,56,63^ TFs regulate genes that control cellular fates,^77–80^ and thus, TFs are being evaluated in clinical trials to treat cancer and chronic wounds, as well as guide tissue regeneration and modulate immune responses.^81^ Previously, we engineered LLO strains to express and deliver signal transducer and activator of transcription 1 (STAT-1) together with Krüppel-like factor 6 (KLF6) for polarization toward a proinflammatory phenotype and Krüppel-like factor 4 (KLF4) together with GATA binding protein 3 (GATA-3) to polarize macrophages toward an anti-inflammatory phenotype.^82–86^ Here, we report that engineered LLO strains, expressing these TFs, altered patterns of BMDM gene expression, cytokine/chemokine expression, and functional metabolism with patterns of modulation toward anti- or proinflammatory phenotypes within a complex response to the bacteria. Furthermore, in murine 4T1 orthotopic breast cancer, the TME^87,88^ was altered by the engineered LLO strains with shifts in the immune cell composition leading to attenuation of tumor growth. Additionally, the safety of this EES platform was observed as multiple doses could be injected without overt effects on the health of mice. By expressing TFs from the EES, macrophage function was modulated demonstrating that this approach is a promising therapeutic strategy and has applications in the study of cell biology (Supporting Information Figure S1 and S2).

## Results

###  LLO Escaped Phagosome Destruction but Was Eliminated by Autophagy

Some of the results and figures in this publication were excerpted with permission from Dr. Madsen’s PhD thesis; these materials have not been published elsewhere.^89^ Fluorescent microscopy confirmed that LLO escaped phagosomal destruction in BMDMs (Figure 1) with spatial overlap of bacteria (white) and LAMP-1-positive structures (phagosomes, magenta).^91^ Phagosome escape was conditional on transcription induction by isopropyl β-d-1-thiogalactopyranoside (IPTG) of the *hlyA* gene encoding LLO, which had also been observed for monocyte/macrophage cell lines.^8,72^ Without IPTG addition (-IPTG), only very few intact rods were observed outside cells or as punctate regions within BMDMs, associated with LAMP-1-positive regions at 4 h (Figure 1, orange arrow). By 12 h, only remnants of bacteria were observed (Figure 1, orange arrowhead). Conversely, when IPTG was added (+IPTG), rods were observed in several cells, not colocalized with LAMP-1 pockets, and some cells contained many bacteria at 4 h (Figure 1, white arrow). Yet, after LLO escaped into the cytoplasm (+IPTG), BMDMs responded and removed most of the intracellular bacteria by 12 h (Figure 1). Microtubule-associated protein light chain 3B (LC3) has been shown to coordinate autophagy response after phagosomal escape in macrophages infected with and other pathogens.^92–95^ LC3B^92^ staining was used to indicate that this mechanism was activated at 12 h by the BMDMs in response to intracellular LLO with some activation and destruction of LLO at 4 h (Figure 1 white arrows). Accordingly, LLO expression, when induced by IPTG, allowed LLO to access the cytoplasm of the BMDMs but resulted in LLO elimination within several hours. Nonetheless, live cell imaging validated that LLO remained viable within the cytoplasm as replication was observed in multiple cells, shown in a representative region of interest, between 3 and 4.5 h post bacterial addition (Supporting Information Figure S3). Additionally, BMDMs were observed to actively pursue and share bacteria between cells to control bacterial proliferation and persistence (Supporting Information Figure S3).

**Figure 1:**
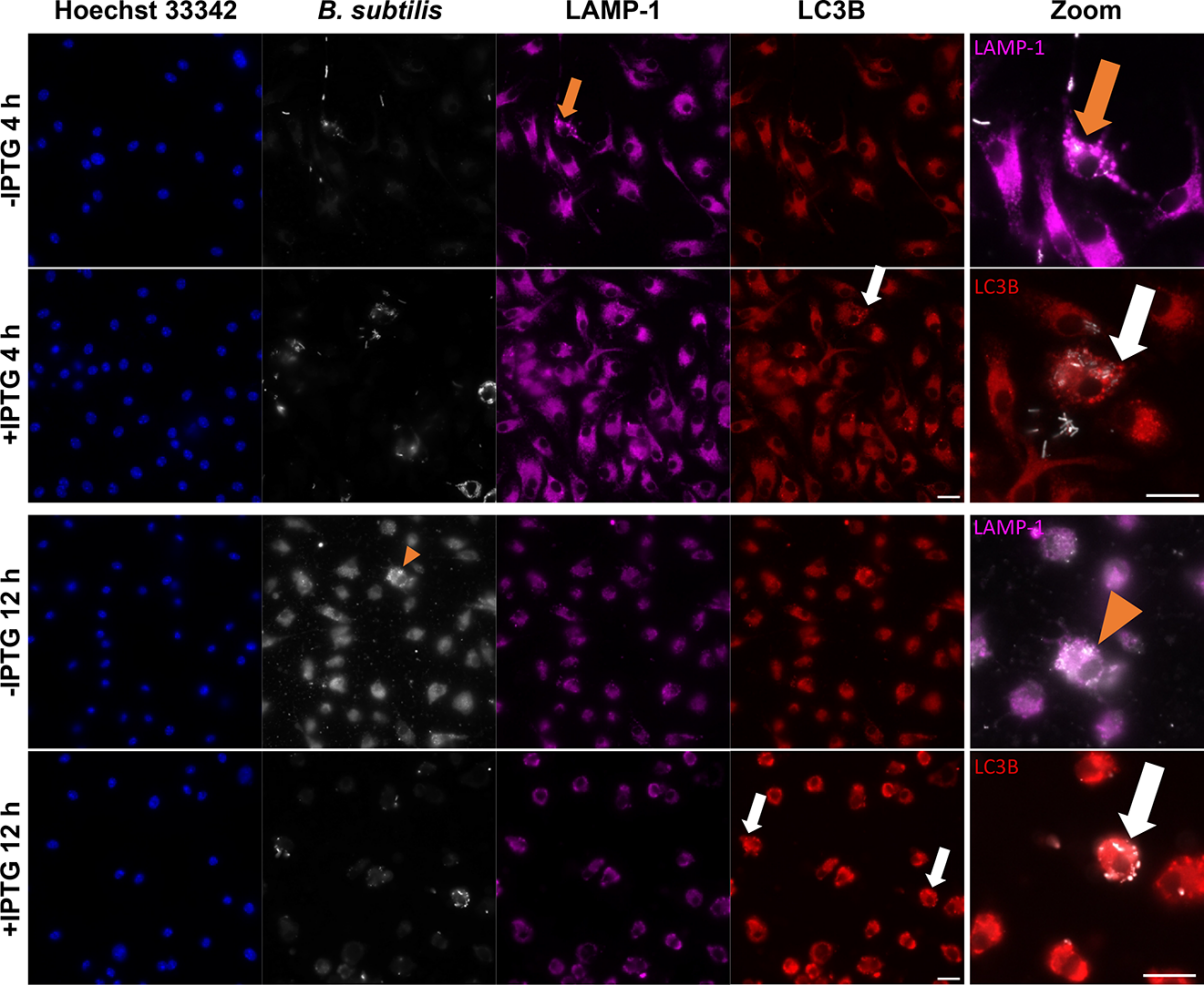
Fluorescent microscopy revealed LLO escape from BMDM phagosomes and destruction by autophagy after uptake was visualized using fluorescent microscopy. BMDM nuclei were stained by Hoechst 33342 (blue), and was stained by CellTracker Orange CMRA Dye (white). LAMP-1 (magenta) and LC3B (red) were used to stain subcellular structures. The LLO strain was delivered to BMDMs at a multiplicity of infection (MOI) of 50:1 and treated without (-IPTG) or with IPTG (+IPTG) with imaging at 4 and 12 h. Zoomed overlays of and LAMP-1 or LC3B are shown (right panel) for -IPTG and +IPTG conditions at 4 h (top) and 12 h (bottom). Without IPTG, there were few -positive regions within cells and were located within LAMP-1-positive pockets in cells at 4 h (orange arrows). With IPTG (+IPTG), -positive regions were present throughout several cells without a strong LAMP-1 signal, but, in some cells, positive LC3B pockets were associated with -positive regions at 4 h (white arrows). By 12 h, only punctate -positive regions were seen in the -IPTG condition, suggesting destruction, with few pockets of LAMP-1 and LC3B (orange arrowhead). However, addition of IPTG resulted -positive regions at 12 h that were all associated with LC3B-positive regions (white arrows). The *z*-depth was chosen for each overlaid image to visualize the center *z*-plane of the BMDMs to show EES localization and relation to protein-labeled mechanisms, and each channel was adjusted to provide a representative image of each scenario. Scale bars = 20 μm.

### TF-Specific Change of BMDM Gene Expression by Engineered LLO Strains

Macrophage gene expression patterns can reveal key behavioral changes. Gene expression is modulated by TFs as part of signaling cascades from various immune stimuli. Therefore, bulk RNA sequencing was performed to elucidate the BMDM gene expression changes in response to engineered LLO strains and controls. Data for all treatments was used to generate a 3D principal component analysis (PCA) emperor plot to visualize genome-wide gene expression changes for all treatments (Supporting Information Figure S4; https://github.com/madsen16/Engineered-endosymbionts). Some figures and text in this section and sections below were excerpted from Dr. Madsen’s PhD thesis; none of these materials have been previously published.^89^ To assess the influence of TF on BMDM gene expression compared to the cytosolic presence of LLO, further PCA analysis was used to compare the TF strains to LLO. The TFs influenced genome-wide BMDM gene expression compared to the shifts caused by LLO (Figure 2). Both LLO *Stat-1Klf6* (LLO-*SK*)^8^ and LLO *Klf4Gata-3* (LLO-*KG*)^8^ altered BMDM gene expression in patterns that were distinguishable compared to the LLO strain alone.

**Figure 2:**
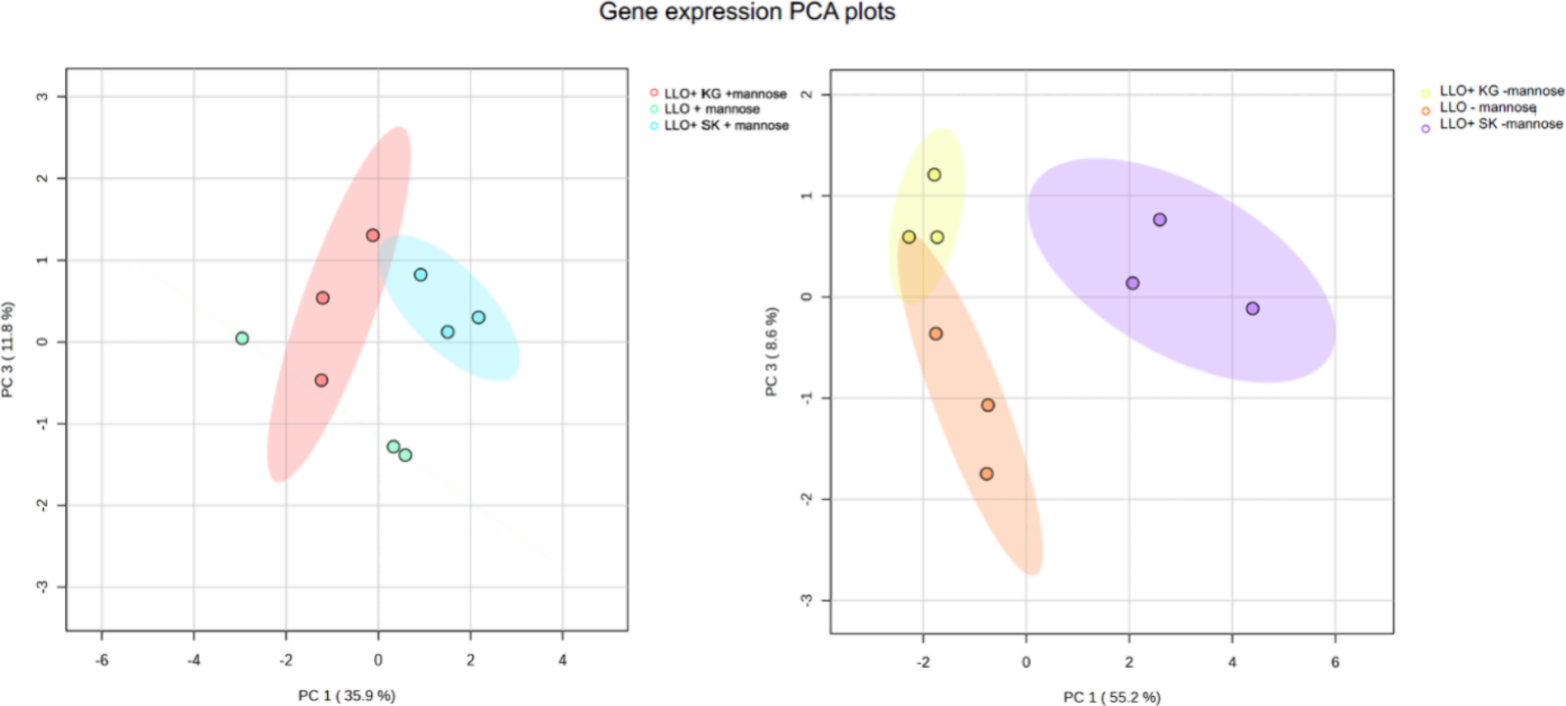
TFs directed BMDM gene expression. PCA plots were used to visualize genome-wide gene expression differences between the following treatments: BMDMs cells treated with the LLO strain, with and without mannose (LLO -mannose, LLO +mannose), LLO-*SK* with and without mannose (LLO-*SK* -mannose, LLO-*SK* +mannose), and LLO-*KG* with and without mannose (LLO-*KG* -mannose, LLO-*KG* +mannose). The 24 h time points are shown. Shaded regions represent 95% confidence intervals.

### Analysis of BMDM Viability, Marker Expression, and Cytokine/Chemokine Production after Exposure to Engineered LLO Strains

Flow cytometry was used to assess efficacy of LLO (LLO strain) internalization into BMDM in vitro when added at different MOI. LLO was stained with CellTracker Orange (CTO) CMRA Dye prior to coincubation with BMDM and added at MOI of 25:1, 50:1, or 100:1 (Supporting Information Figure S5). Cells, which had CTO signal (%BMDM and alive), were identified as those containing LLO, and the various MOI resulted in 16, 33, and 29% positive cells. BMDM viability was also assessed by flow cytometry at an MOI of 50:1 at 4 and 12 h, either with or without LLO induction (±IPTG). After 4 h of incubation with the bacteria, IPTG induction of LLO caused a 5% loss of total cells, with no significant difference in cell numbers at 12 h. When there was no LLO induction, no loss in BMDM viability was observed (Supporting Information Figure S5).

BMDM surface marker expression of CD11b+ F4/80+ was evaluated by flow cytometry to identify shifts in macrophage function caused by engineered LLO strains at 24 and 48 h. BMDMs were incubated with the LLO strain LLO-*SK* or LLO-*KG*. For comparison, BMDMs were incubated in parallel with LPS and IFN-γ as a control for a proinflammatory phenotype and IL-4 and IL-13 as an anti-inflammatory phenotype control. These were marked by expression differentiation markers CD86 and CD206.^18,96,97^ For CD86, LPS and IFN-γ increased marker expression by 15- and 9-fold at 24 and 48 h, respectively (Supporting Information Figure S6). There was a significant difference in CD86 expression among all treatment groups at both 24 and 48 h time points (*p* = .016, Supporting Information Figure S6). BMDMs treated with all bacterial conditions resulted in a trend of increased (4.92-fold mean) CD86 expression at 24 h when compared to untreated BMDM. At 48 h, there was no significant increase in CD86 expression in any of the bacterial-treated groups. However, LLO-*KG* ±mannose-treated groups had the greatest decrease in CD86 expression (average 2.7-fold less) versus LLO-*SK* ±mannose with the highest increase in CD86 expression (average 1.1-fold) when compared to untreated BMDM. For CD206, IL-4 and IL-13 increased marker expression by 6- and 2-fold at 24 and 48 h, respectively (Supporting Information Figure S6). At 24 h, there was no significant difference in CD206 expression in any bacteria-treated groups compared to untreated BMDMs. At 48 h, all bacterial conditions caused a significant decrease in the level of CD206 expression. There was no difference in CD206 expression between LLO strains, but there were increases in CD206 expression when mannose was added in the LLO (*p* = .101), LLO-*SK* (*p* = .011), and LLO-*KG* (*p* = .0008) groups.

As key mediators of immune cell signaling, cytokines and chemokines were profiled in BMDM cultures to support translation to in vivo.^15,17^ BMDM cultures secreted significantly increased amounts of cytokines and chemokines in response to the bacterial treatments (several hundred or thousand-fold in some instances) and in many cases more than the positive controls, with LLO-*SK* and LLO-*KG* leading to different levels in the cultures (Figure 3, Supporting Information Figure S7). LLO-*SK* and LLO-*KG* led to different levels of IL-10, macrophage inflammatory protein-1α (MIP-1α), and granulocyte colony-stimulating factor (G-CSF) especially when TF expression was induced from the LLO strains by mannose; this was observed in comparison to each other and when compared to the LLO strain at 24 h (Figure 3a,e,f). The LLO-*KG* increased IL-6 levels in comparison to those of LLO-*SK* and the LLO strain at 24 h (Figure 3b). Tumor necrosis factor α (TNF-α) increased with all bacterial treatments at 24 h, but at 48 h, only the LLO-*SK* strain with mannose significantly increased TNF-α in comparison to the untreated BMDMs and comparable to that of the positive control (Figure 3c,d). MIP-2 (CXCL2) and monocyte chemoattractant protein-1 (MCP-1/CCL2) showed specific responses to d-mannose with the different engineered strains still causing different patterns, even with the complexity of addition of d-mannose at 24 h (Figure 3g,h). d-Mannose continued to impact the levels of these two proteins at 48 h (Supporting Information Figure S7e,f). While production of most cytokines and chemokines was triggered more by the bacteria than the positive controls, IL-12p40 and vascular endothelial growth factor (VEGF) did not show these trends. Production of both IL-12p40 and VEGF at 24 and 48 h increased in response to the proinflammatory positive control by either hundreds- or tens-fold, respectively (Supporting Information Figure S7m–p). The bacteria did not cause such large increases. Yet, at 24 h, IL-12p40 exhibited differential regulation by LLO-*SK* and LLO-*KG;* this was observed previously with LLO-*KG* increasing production by 5-fold in comparison to the untreated cells.^8^ However, many of the cytokines and chemokines such as IL-10, IL-6, and MCP-1 are examples that did not show any differences in the bacterial treatments at 48 h (Supporting Information Figure S7a–f). Furthermore, some of the cytokines such as IL-15 and granulocyte-macrophage colony-stimulating factor (GM-CSF) were not significantly produced in response to any treatments (Supporting Information Figure S7g,h,k,l). Finally, IL-1β was significantly produced only in response to the bacterial treatments at both 24 and 48 h, but no production difference was observed between the bacterial treatments (Supporting Information Figure S7i,j).

**Figure 3:**
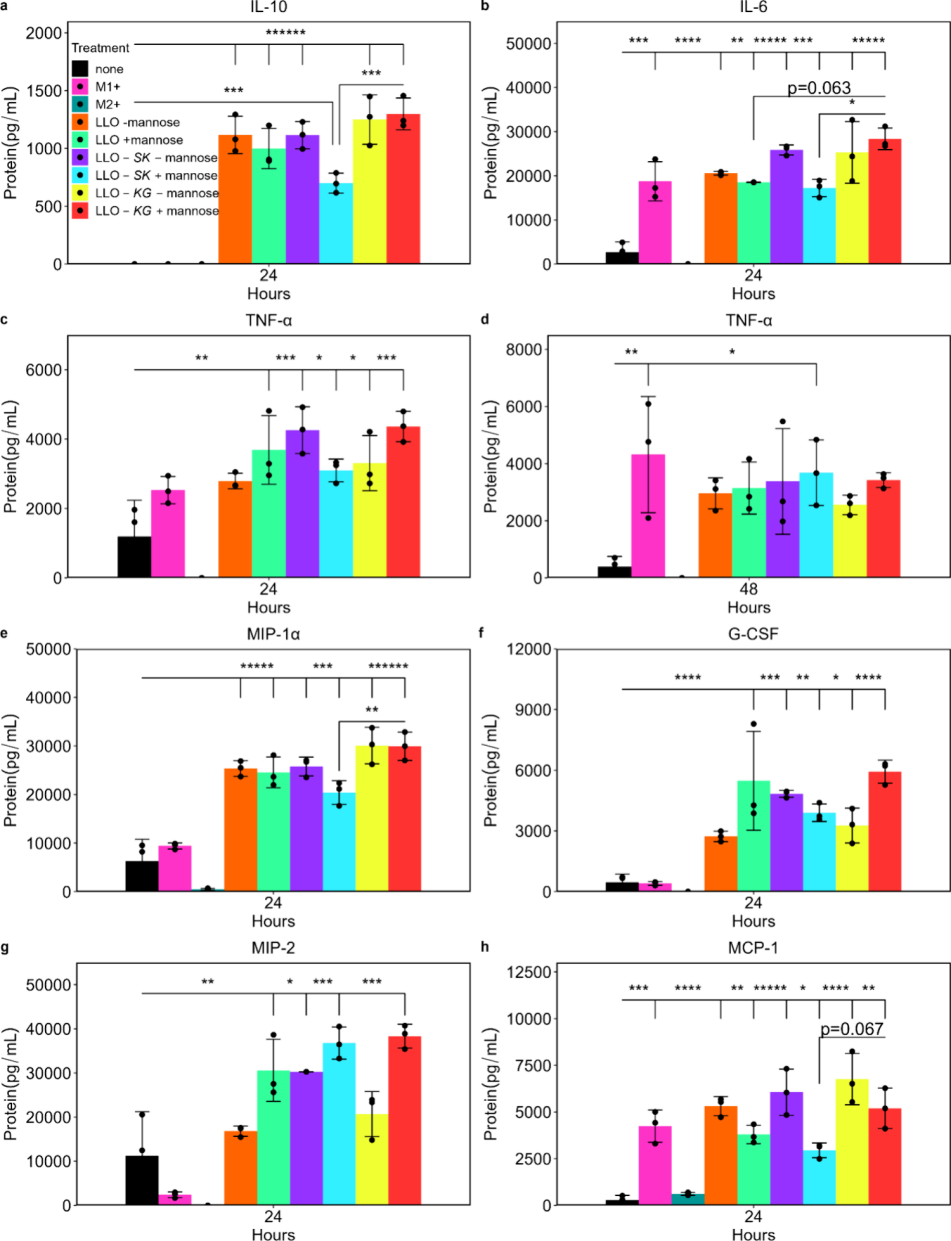
Engineered LLO strains altered BMDMs cytokine and chemokine production. Cytokine and chemokine protein concentration was quantified in the media after BMDM cells were untreated (none), treated with LPS and IFN-γ (pro-inflammatory, pink), IL-4 and IL-13 (anti-inflammatory, green), LLO strain with and without mannose (LLO -mannose, orange, LLO +mannose, light green), LLO-*SK* with and without mannose (LLO-*SK* -mannose, purple, LLO-*SK* +mannose, blue), and LLO-*KG* with and without mannose (LLO-*KG* -mannose, yellow, LLO-*KG* +mannose, red) at 24 and 48 h postinitial treatment. IPTG was added to all of the bacterial treatments. Data for (a) IL-10 at 24 h, (b) IL-6 at 24 h, (c, d) TNFα at 24 and 48 h, (e) MIP- 1α at 24 h, (f) G-CSF at 24 h, (g) MIP-2, and (h) MCP-1 at 24 h are shown. The data is mean ± SD from *n* = 3 biological replicates; *p < 0.05, **p < 0.01, ***p < 0.001, ****p < 0.0001, *****p < 0.00001, ******p < 0.000001.

### Engineered LLO Strains Affect Functional Metabolism Patterns in BMDMs Maintained In Vitro

Functional metabolism provides valuable insight into the activity and response of macrophages to various stimuli.^98^ Additionally, d-mannose has been shown to impact macrophage metabolism and function;^99^ therefore, its effects were considered in this study. BMDM functional metabolism was characterized in response to the engineered LLO strains and mannose with LPS serving as a control, and these were compared to prior studies.^21,22^ Overall, the d-mannose and engineered LLO strains modified BMDM functional metabolism in unique patterns at both 12 and 24 h as indicated by OCR, ECAR, and ATP production as indicators of shifts between glycolysis and oxidative phosphorylation (Figure 4, Supporting Information Figure S8, Tables 1 and 2). The bacterial treatments increased basal OCR and ECAR similar to the LPS treatment at 12 h, while results of d-mannose treatment were similar to no treatment (Figure 4c,d). The LLO-*KG* strain, with and without mannose, increased basal OCR and ECAR more than any other condition at 12 h (Figure 4c,d). Conversely, by 24 h, d-mannose overtook all other treatments as the dominating stimulus driving the change in metabolism even in the bacterial treatments (Supporting Information Figure S8c,d). The LLO-*KG* strain still caused trends of increased levels of OCR and ECAR but only without the addition of d-mannose.

**Figure 4:**
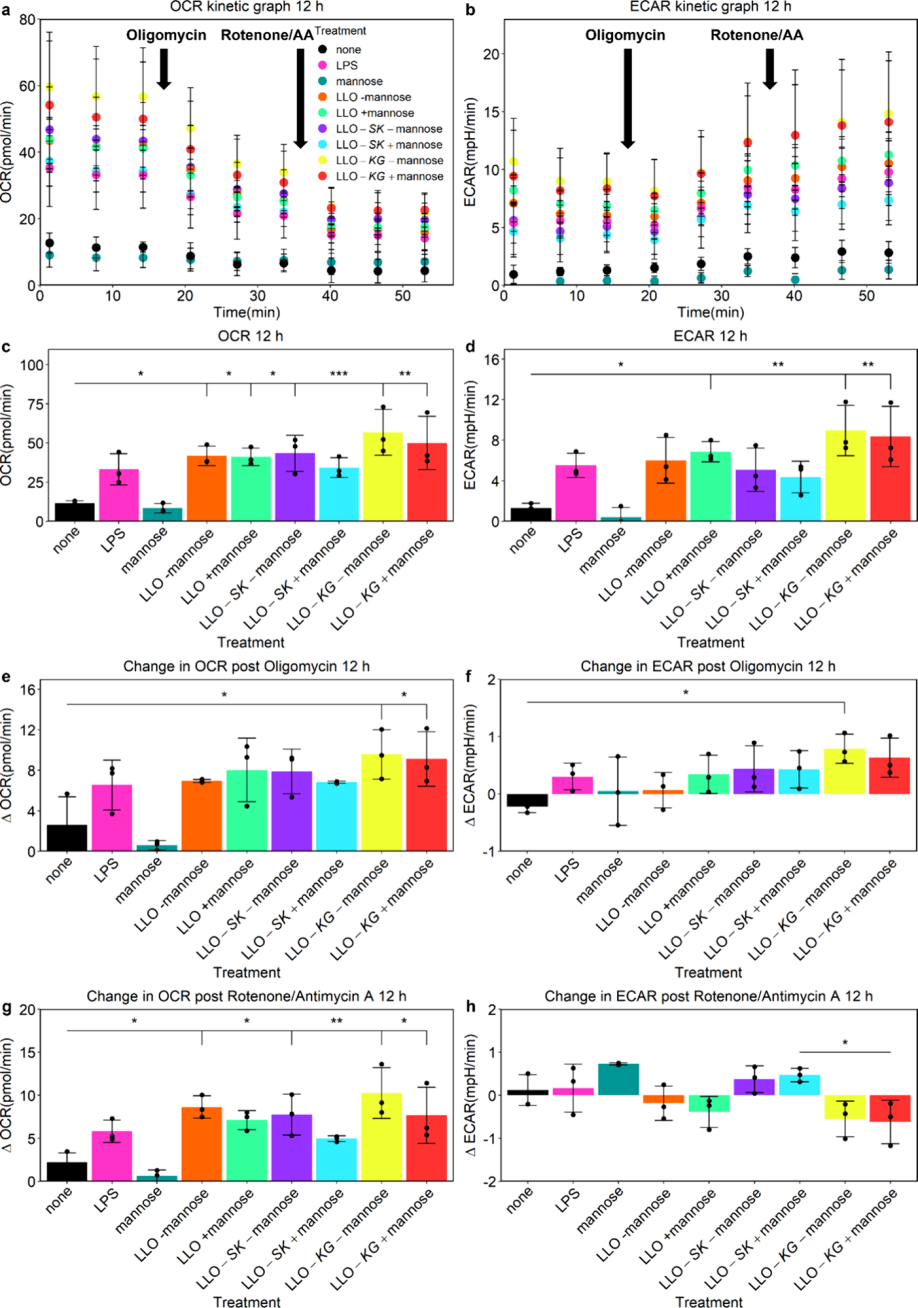
Shifts in functional metabolism patterns of BMDMs at 12 h after exposure to engineered LLO strains. OCR and ECAR were measured before and after addition of the electron transport chain inhibitors, oligomycin and rotenone/antimycin A (AA), which were added at points indicated on kinetic plots (a, b). Basal OCR and ECAR quantification at the third measurement before addition of inhibitors were plotted (c, d). Further analysis was performed to quantify changes in the levels of the OCR (ΔOCR) and ECAR (ΔECAR) after the inhibitors were added (e–h). BMDMs were untreated (none), treated with LPS, mannose, LLO strain with and without mannose (LLO -mannose, LLO +mannose), LLO-*SK* with and without mannose (LLO-*SK* -mannose, LLO-*SK* +mannose) and LLO-*KG* with and without mannose (LLO-*KG* -mannose, LLO-*KG* +mannose). IPTG was added to all bacterial treatments. Data is mean ± SD from *n* = 3 biological replicates; *p < 0.05, **p < 0.01, ***p < 0.001.

To further analyze responses to the bacteria alone and how the TFs may be impacting changes in metabolism, levels of OCR (ΔOCR) and ECAR (ΔECAR) were evaluated when the electron transport chain inhibitors oligomycin (complex V) and rotenone (complex I)/antimycin A (AA; complex III) were added.^100^ At 12 h, LLO-*KG* with and without d-mannose caused a significant change in OCR when oligomycin was added, and rotenone/AA treatment resulted in similar results as basal OCR (Figure 4e,g). With changes in ECAR at 12 h, the addition of rotenone/AA revealed different trends between the LLO-*SK* and LLO-*KG* strains after d-mannose treatment (Figure 4h). At 24 h, d-mannose was the dominant stimulus when evaluating changes in the level of the OCR after inhibitors were sequentially added (Supporting Information Figure S8e,g). However, the changes in ECAR at 24 h after addition exhibited less dominance by d-mannose and even some differential trends between the engineered LLO strains when oligomycin was added (Supporting Information Figure S8f,h).

Quantification of the energy generation contribution from glycolysis or oxidative phosphorylation demonstrated that the bacterial treatments shifted the metabolism to glycolysis from oxidative phosphorylation at 12 h (Supporting Information Table S1). The LLO-*KG* strain with mannose resulted in the most significant shift to glycolysis (*p* < 0.007) comparable to that of LPS (*p* < 0.005) while LLO-*SK* with mannose did not produce a significant shift. Additionally, total ATP production rates followed the same trends as the LLO-*KG* and LLO strain +mannose, which increased ATP rates by 4-fold (*p* < 0.01) and 3-fold (*p* < 0.07), respectively, compared to the untreated cells at 12 h. d-Mannose diverted all energy production to oxidative phosphorylation and led to a reduced ATP production rate at 12 h (Supporting Information Table S1). Energetic contribution from either glycolysis or oxidative phosphorylation also paralleled the results of the OCR and ECAR at 24 h except in the LLO-*KG* with mannose treatment (Supporting Information Table S2). The treatment of d-mannose at 24 h shifted energy generation toward glycolysis instead of oxidative phosphorylation, which was inverse of trends at 12 h. The LLO-*KG* strain inhibited that shift when treated with d-mannose, which was similar to the case for the other bacterial treatments without d-mannose. In contrast, treatment with the LLO strain and LLO-*SK* strain were significantly affected by the addition of d-mannose, *p* < 0.04 and *p* < 0.004, respectively (Supporting Information Table S2). However, ATP production rates appeared to be largely driven by d-mannose in all bacterial treatments and were similar to those of the d-mannose treatment alone.

### Tumor Growth Rate Decreased by Engineered LLO Strains without Overt Negative Health Effects on Mice

Cancer bacteriotherapies have been shown to be most effective at reducing tumor growth and altering the tumor environment when injected intratumorally (IT).^53,54,69,101^ However, if the bacteria could be injected intravenously (IV) and were to naturally concentrate in the TME, this would facilitate clinical translation.^102,103^ Therefore, we explored whether the engineered LLO strains would accumulate in the TME after IV injection and alter the TME. Bioluminescent, nonpathogenic LLO-*luxA-E* ^104,105^ (LLO-*lux*) was shown to localize to 4T1 orthotopic tumors and persist in the tumor for a week after IV injection of 10^8^ bacteria while being cleared from healthy BALB/c mice in 24 h (Supporting Information Figure S9c,e,f). Furthermore, immunohistochemistry of excised tumor sections revealed Gram-positive structures (purple; black arrowheads) in the same spatial location as both phagocytes (CD11b+; brown; black arrow) and other CD11b- cells in the tumor (Supporting Information Figure S9g,h). Moving through the *z*-depth allowed for visualization of Gram-positive structures, which were clumped together within cells. Tumor sections from mice, which did not receive a bacterial injection, did not have any detectable bacteria (Supporting Information Figure S9i). Accordingly, the effect on the progression of tumor growth was measured after the engineered LLO strains were injected. Animals received IV injections once a week for 2 weeks after which all mice were sacrificed and tumors were analyzed by TME immunophenotyping. LLO-*SK* +mannose caused the greatest reduction in normalized tumor growth (2.5-fold) relative to all other treatments, and in comparison, to the untreated animals, which was shown to be TF specific because all CFU counts were similar in all tumors (Supporting Information Figure S10). Furthermore, LLO-*SK* +mannose caused significant reduction in tumor growth as early as 3 days postinitial injection (Figure 5). Also, LLO-*SK* +mannose significantly reduced tumor growth compared to LLO-*KG* +mannose, which reduced tumor growth by half in comparison to the untreated tumors (Figure 5). In a separate group, LLO-*SK* was injected with both IV (once a week) and IT (every third day between the weekly IV) to test for increased efficacy. LLO-*SK* IV+IT did not further reduce tumor growth in comparison to IV only (LLO-*SK;* Figure 5). d-Mannose alone also decreased tumor growth compared to the untreated tumors (Figure 5, *p* < 0.000001). When d-mannose was added to all bacterial treatments, only the TF strains showed a significant further reduction in tumor growth compared to the d-mannose treatment alone, while the LLO-*lux* strain did not (Figure 5).

**Figure 5:**
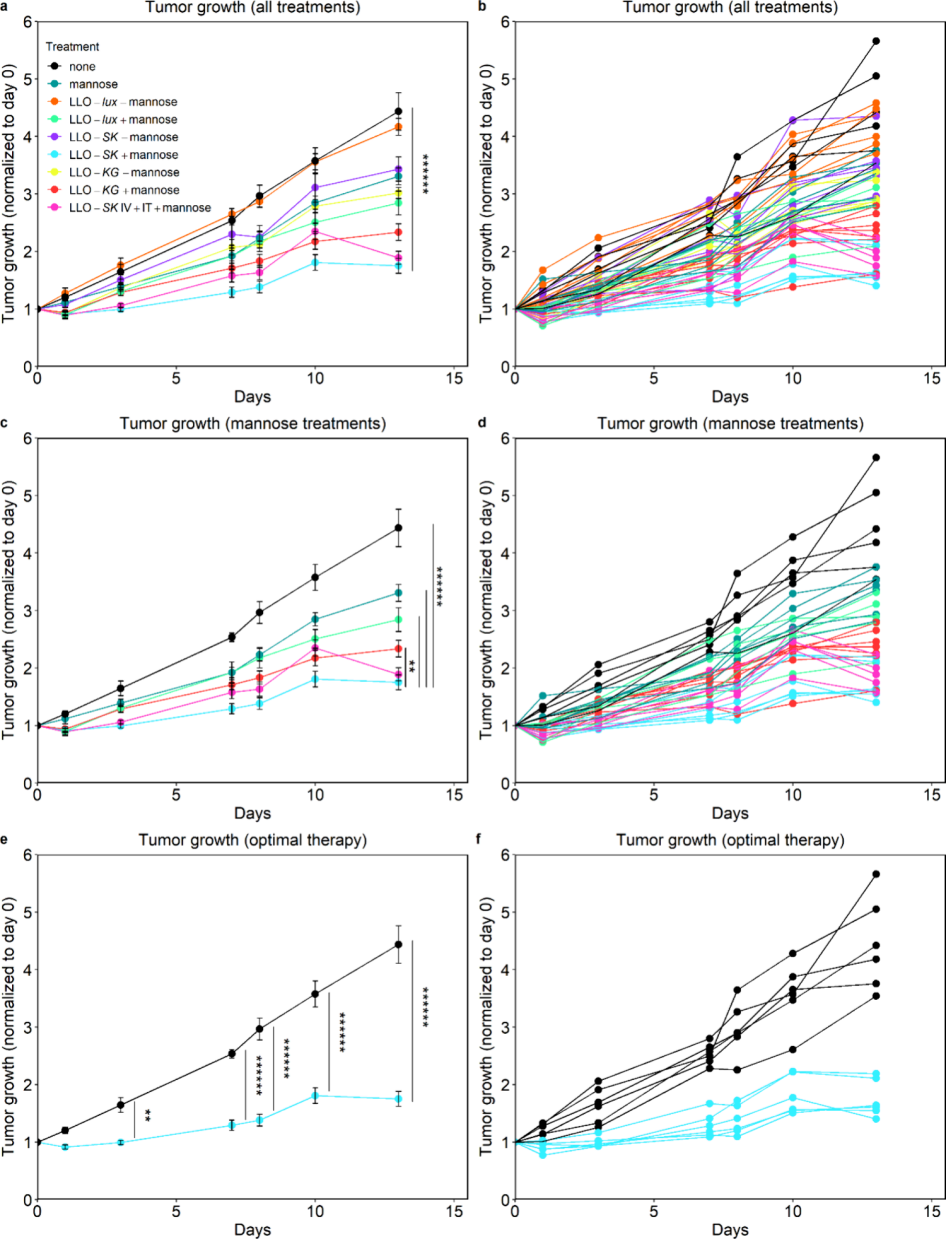
Bacteria-mediated reduction of tumor growth. (a) Average tumor growth normalized (to day of bacterial injection, day 0) for all groups is shown and (b) also for all animals in the study, (c) for groups of animals treated with mannose, (d) for individual animals treated with mannose, and last, (e, f) for groups of control (black) and LLO-SK treated mice (blue). Tumors were untreated (none), treated with mannose, LLO-*lux* strain injected IV with and without mannose (LLO-*lux* -mannose, LLO-*lux* +mannose), LLO-*SK* injected IV with and without mannose (LLO-*SK* -mannose, LLO-*SK* +mannose), LLO-*KG* injected IV with and without mannose (LLO-*KG* -mannose, LLO-*KG* +mannose), and LLO-*SK* injected IV and IT with mannose (LLO-*SK* IV+IT +mannose). IPTG was added to all bacterial treatments. (a, c, e) Data is mean ± SEM from *n* = 6 mice; **p < 0.01, ******p < 0.000001.

To investigate the health effects on the mice, mouse body mass was measured throughout the experiment, and liver histopathology was used at the end of the experiment to determine any side effects in the liver. After intravenous injection of strains alone, there was no reduction in mouse body mass over the 13 day period. Only when d-mannose was added to drinking water did we observe a decrease in mouse body mass beginning at day 3. When compared to the untreated group, +mannose (*p* = 0.013), LLO +mannose (*p* = 0.001), LLO-*KG* +mannose (*p* = 0.034), and LLO-*SK* IV+IT (*p* = 0.011) groups had mouse body masses that were significantly lower at the end point but not the mice in the LLO-*SK* +mannose group (Supporting Information Figure S11). The group that lost the most weight by day 13 was the group that received mannose only (−9.2% loss compared to the beginning of treatment). Liver histopathology did not identify any differences between untreated and bacteria-treated groups. Within all groups, there were multifocal perivascular and random mononuclear and neutrophilic cell infiltrates, and any other findings were normal within a physiological range of individual animals (Supporting Information Table S3). Bacteria were not identified in the liver by light microscopy in any group following Giemsa staining. Additionally, after LLO-*lux* was injected into healthy mice, we observed zero CFUs in either the livers or the spleens of healthy, nontumor bearing, mice (Supporting Information Figure S10).

### Tumor Immunophenotyping and Metabolism Identified Immune Cell Population and Functional Changes Caused by Engineered LLO Strains

Profiling of the tumor immune cell populations to determine alterations to the TME showed that the engineered LLO strains can alter immune cell populations in tumors (Figure 6, Supporting Information Figure S12). While there was no significant change in the total number of CD11b+ myeloid cells with bacterial treatment, when compared to the untreated group, bacterial treatments did decrease the number of monocytic myeloid-derived suppressor cells (M-MDSC; CD45^+^CD11b^+^Ly6C^high^Ly6G^–^) including treatments with LLO-*SK* multiple injections +mannose (*p* = 0.0003) and LLO +mannose (*p* = 0.032). Granulocytic myeloid-derived suppressor cells (G-MDSC; CD45^+^CD11b^+^Ly6G^+^Ly6G^low^) were decreased in the LLO-*SK* +mannose group when compared to the +mannose only group (*p* = 0.045) and LLO +mannose group (*p* = 0.028). Anti-inflammatory tumor-associated macrophages (TAMs; CD45^+^CD11b^+^Ly6G^–^Ly6C^low^CD206^+^) decreased in the LLO-*SK* multiple injection +mannose group (*p* <.0001) and LLO +mannose group (*p* = 0.041), which highlighted the ability to decrease these cell populations that are key players in tumor progression and contribute to a protumoral phenotype. Further, the LLO-*SK* multiple treatment group significantly increased the proinflammatory/anti-inflammatory TAM ratio compared to all other treatments, except LLO +mannose (*p* = 0.086). Mature regulatory T cells (Tregs; CD25^+^FoxP3^+^) are another cell type known to suppress immune response. Their presence was slightly decreased in the LLO-*SK* +mannose-treated group (*p* = 0.096) when compared to the untreated group. Furthermore, alternative FoxP3+CD25- populations, which are thought to have no suppressive function, were increased in the LLO-*SK* multiple injection group when compared to the untreated group (*p* = 0.048).^106^ Tregs in the classical preactivation state (FoxP3-CD25+) were decreased in the LLO-*SK* multiple injection group when compared to untreated (*p* = 0.052) and the LLO +mannose group (*p* = 0.047). It was seen that the introduction of LLO (without transcription factors or mannose) did not initiate a significant change in any immune cell population when compared with the untreated group. The introduction of mannose only influenced M MDSC (*p* < 0.0001), when compared to untreated mice. There were no significant differences between LLO -mannose and LLO +mannose treatment groups in any of the analyzed immune cell populations. There were some changes in immune cell populations that were not expected; LLO-*SK* ±mannose and LLO-*KG* ±mannose all increased anti-inflammatory TAMs when compared to LLO +mannose. In addition, LLO-*SK* multiple injections decreased the dendritic cell (DC) population compared to untreated mice (Figure 6).

**Figure 6:**
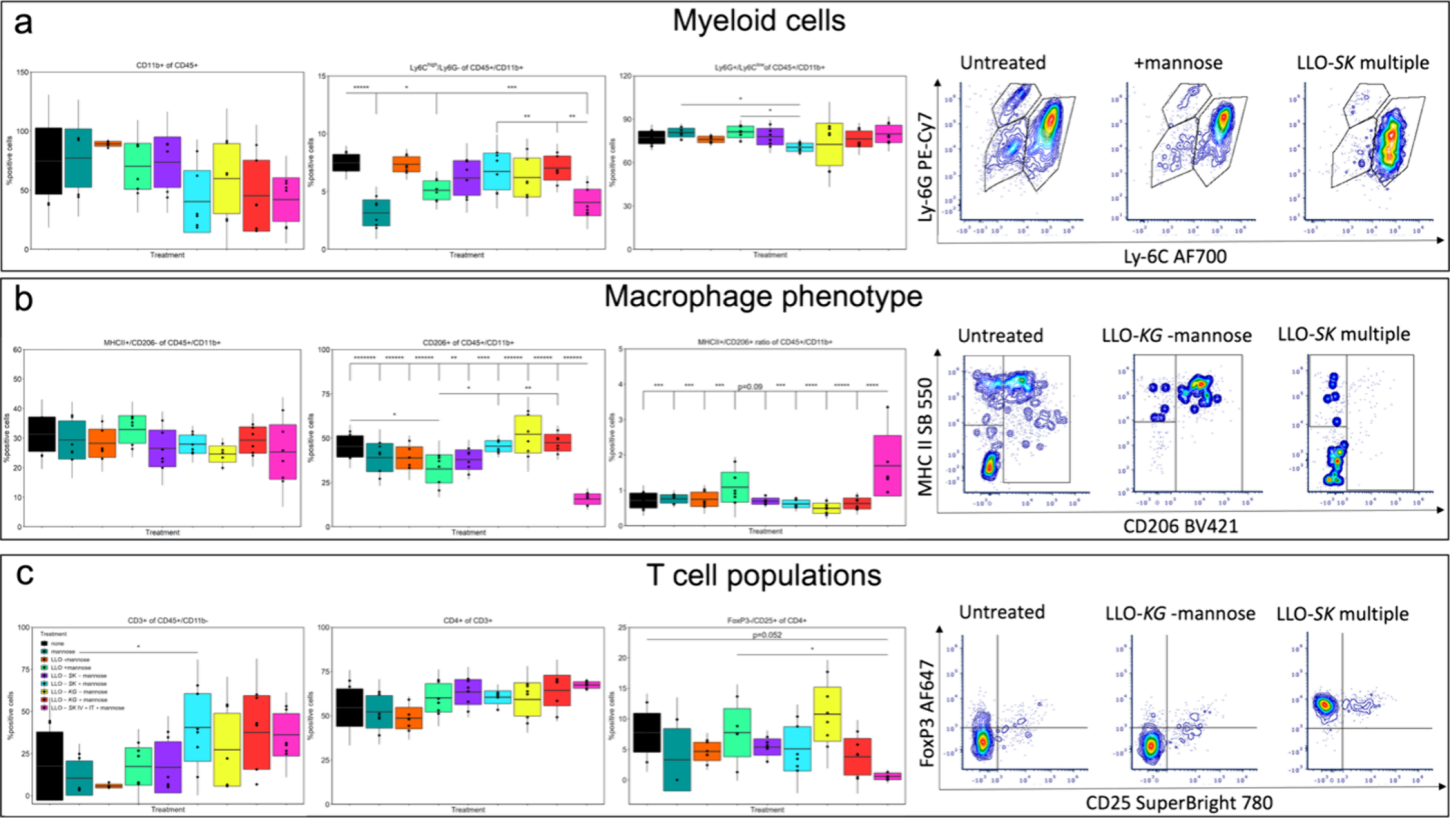
Tumor immunophenotyping revealed modifications in tumor immune cell populations. Immune cell populations were analyzed using flow cytometry on cells from dissociated tumors in all treatment groups: untreated (none), treated with mannose, LLO-*lux* strain injected IV with and without mannose (LLO-*lux* -mannose, LLO-*lux* +mannose), LLO-*SK* injected IV with and without mannose (LLO-*SK* -mannose, LLO-*SK* +mannose), LLO-*KG* injected IV with and without mannose (LLO-*KG* -mannose, LLO-*KG* +mannose), and LLO-*SK* injected IV and IT with mannose (LLO-*SK* IV+IT +mannose). Profiles for (a) myeloid, (b) macrophages, and (c) T cells are shown. Populations analyzed were myeloid cells (CD11b^+^ of CD45^+^), monocytic myeloid-derived suppressor cells (M MDSC; Ly6C^high^/Ly6G^–^ of CD11b^+^), granulocytic MDSC (G MDSC; Ly6G^+^/Ly6C^low^ of CD11b^+^), proinflammatory macrophages (MHCII^+^/CD206^–^ of CD11b^+^), anti-inflammatory macrophages (CD206^+^ of CD11b^+^), anti-inflammatory/proinflammatory population ratio, T cells (CD3^+^ of CD11b^+^), helper T cells (CD4^+^ of CD3^+^), and an alternative T regulatory cell subset (FoxP3^–^/CD25^+^ of CD4^+^). Data is mean ± SD for box and two times ± SD for whiskers from *n* = 3 tumors in technical duplicates for each treatment; *p < 0.05, **p < 0.01, ***p < 0.001, ****p < 0.0001, *****p < 0.00001, ******p < 0.000001.

Beyond profiling the immune cell populations, metabolism can be used to characterize changes to the TME as metabolism has been linked to cancer progression and to enhancing metastasis.^24,25,100^ Combining this knowledge with the results that the engineered LLO strains could modulate functional metabolism, the TME metabolism was characterized.^100^ We could associate mitochondrial bioenergetics within immune cell populations between individual mice in a treatment group and observed the same trend across the groups. Both the LLO-*SK* +mannose and LLO-*KG* -mannose groups had the highest association between increased percent of CD3+ T cells and increased basal OCR (*R* ^2^ = 0.889, *p* = 0.217, and *R* ^2^ = 0.765, *p* = 0.323, respectively; Supporting Information Figure S13). The group treated with mannose only did not show any correlation (*R* ^2^ = 0.002, *p* = 0.975), suggesting that the presence of mannose in these groups did not have any effect on immunometabolism. A similar trend was observed in other measures such as max respiration as the LLO-*SK* +mannose treatment had the highest association between increased max respiration and increased CD3+ T cells compared to other treatments such as LLO-*KG* -mannose and mannose (*R* ^2^ = 0.898, *p* = 0.207 and *R* ^2^ = 0.82, *p* = 0.279, *R* ^2^ = 0.284, *p* = 0.689, respectively).

## Discussion

For the EES platform to be effective at altering macrophage function and be used in cancer treatment, the EES must interact with the host cells and provide intracellular delivery of TFs. LLO gained access to the cytoplasm of BMDMs after IPTG induction of LLO expression (Figure 1), and the cells were viable (Supporting Information Figure S3); this allowed for intracellular delivery of TFs. Even with destruction by LC3-related autophagy mechanisms^92–95^ bacteria persisted for several hours, which seemed to be sufficient time for TF delivery, and perhaps after destruction could release TFs. Furthermore, the natural removal of the bacteria suggests the safety of the EES platform when used as a therapy. Yet, if use of EES as a therapy requires more prolonged interaction between the host cell and engineered LLO strains, proteins such as phospholipases (plc) A and B from could be introduced into the LLO chassis to evade the autophagy,^95^ but extended replication would need to be considered. After confirmed escape, replication, and destruction by LC3 mechanisms of LLO, the viability of BMDMs remained high (∼95%), suggesting that macrophage function could be altered without loss of host cells (Supporting Information Figure S5). Yet, the incidence of uptake into BMDMs of approximately 35% resulted in an important consideration for other in vitro characterization and in vivo evaluation (Supporting Information Figure S5). The consideration included whether engineered LLO strains delivering TFs to 35% of the host cell population would result in functional change throughout the population and would result in efficacy in vivo. Accordingly, the results seen throughout the study indicated that the cellular function, when measured in populations, was uniquely altered by the different bacterial strains.

Once the EES are in the cytoplasm, effective delivery of TFs, or other payloads designed to alter host cell gene expression, to the appropriate subcellular compartment is essential for the functional change. The engineered LLO strains changed BMDM gene expression in patterns consistent with effective TF delivery, even when compared to the stimulus of the LLO strain in the cytoplasm (Figure 2). Overlap in genome-wide gene expression was observed more in the mannose conditions of the engineered LLO strains because of the effect of mannose on the BMDMs, which has been demonstrated previously.^99^ This observation is further supported by the conditions without mannose having further separation in these data plots, even without bacterial transcription of the TFs being fully induced. Previously, the engineered LLO strains were shown to deliver TFs without transcription fully induced likely due to transcriptional leak in the mannose regulatory system^8,107^ and the partial effects of the TFs were still observed as indicated by alterations in gene expression patterns. Further in-depth analysis should be performed in future studies to understand what regulatory mechanisms and pathways are influenced by EES and TF delivery, especially on a temporal basis. BMDMs are known to have dynamic gene expression that responds in a temporal fashion (as quickly as 1 h or could be 12 to 24 h post stimulus) to simple stimuli such as LPS, which adds more complexity, and studying the more complex EES stimuli in a temporal fashion could provide more insights into how the BMDMs are modulated by different strains.^16^ Additionally, more comparative analysis could be performed among all the treatment groups to assess new target pathways to be used in influencing macrophage behavior through new TF delivery or other molecules of interest (Supporting Information Figure S4).

Differential BMDM marker expression, cytokine/chemokine production, and rates of metabolism provide a profile of functional changes in macrophage function and an overview of the impact on the BMDMs by the bacteria and the TFs. The SK TFs cooperate to generally promote an antitumoral, proinflammatory macrophage phenotype. STAT1 functions downstream of IFN receptors to drive the transcription of immune-activating genes^80,108^ while KLF6 supports M1 polarization by cooperating with NF-κB and suppressing M2 macrophage differentiation through inhibition of PPAR-γ signaling.^85^ In contrast, the KG TFs generally contribute to a protumoral, anti-inflammatory phenotype, though these factors together have been observed to have pleiotropic behavior, as do many TFs depending on initial stimulus and signaling from other cells. Typically, both act downstream of STAT6 and promote M2 gene expression by binding to the promoters of M2-associated genes, while concurrently repressing M1-associated inflammatory pathways.^86,109^ The lack of CD86 expression in response to the LLO from the BMDMs was unexpected because Toll-like receptor 2 (TLR2) triggers inflammatory responses,^110,111^ but a similar lack of CD86 marker expression has been observed when bacterial TLR agonists were used to stimulate initially resting BMDMs, which was also observed for CD206.^112^ Unstimulated BMDMs appeared to produce a large response in cytokines but did not change marker expression unless preactivation was performed before addition of the bacterial TLR agonists.^112^ Further, the CD206 expression response was more complex because CD206 is the mannose-binding receptor^113–115^ and can act as a receptor for bacterial surface carbohydrates.^116^ Cytokines and chemokines play essential roles in signaling between various immune cell populations,^17,38,117,118^ and these proteins have been shown to exhibit pleiotropic effects in disease progression and in cell classification.^33,119–125^ Yet, unraveling this complexity provides valuable insights into outcomes in vivo and understanding of therapeutic potential. The engineered LLO strains stimulated production of many tumor relevant cytokines and chemokines from the BMDMs with the TFs causing specific alterations presumably due to changes in gene regulation (Figure 3, Supporting Information Figure S7). LLO-*SK* and LLO-*KG* differentially regulated IL-10, MIP-1α, G-CSF, and IL-12p40 at 24 h with IL-10, G-CSF, and IL-12p40 occurring in a predicted pattern based on known regulation.^8,84,126–129^ Further examples of predicted regulation include both LLO-*KG* upregulating IL-6 at 24 h^130^ and LLO-*SK* downregulating MCP-1.^131,132^ d-Mannose appeared to have a similar but opposite effect on MIP-2 as on MCP-1 and all treatments with d-mannose upregulated MIP-2 indicating that d-mannose plays a role in macrophage response. Overall, the engineered LLO strains generated production of beneficial cytokines and chemokines for altering the TME including TNF-α and IL-12p40,^129,133,134^ but the pleiotropic effects of cytokines and chemokines such as IL-6 and MCP-1 add ambiguity as both are implicated in promoting T cell invasion into the TME but can also promote tumor angiogenesis when expressed by cancer cells.^131,135^ Yet, the LLO-*SK* strain did downregulate some cytokines and chemokines implicated in poor tumor outcomes such as MIP-1α, MIP-2, G-CSF, and IL-10 which is beneficial.^127,136–139^

With the complexities seen in marker and cytokine/chemokine expression, functional metabolism was measured to provide another indicator of macrophage phenotype in response to the engineered LLO strains and its relevance in tumors. In totality, the bacterial treatments increased glycolytic flux, similar to LPS treatment.^20–23,26,140^ The LLO-*KG* strain promoted the most significant shift in metabolism with increased levels of OCR, ECAR, and ATP production (Figure 4), likely due to the activity of KLF4 and GATA-3. Both TFs are involved in macrophage metabolism and work with STAT-6 to induce nuclear peroxisome receptor proliferator-activated receptor γ (PPARγ) causing mitochondrial biogenesis, which could result in increased OCR and ATP production rate.^86,98,141,142^ Additionally, OCR, an index of oxidative phosphorylation, can be simultaneously elevated with glycolytic flux during inflammation, being linked to production of cytokines and chemokines, such as IL-10, IL-6, and TNF-α.^141^ These complex regulatory networks likely explain how the LLO-*KG* strain significantly increased oxidative phosphorylation and glycolysis at 12 h and then was able to diminish a shift from oxidative phosphorylation to glycolysis caused by d-mannose at 24 h. Uniquely, OCR in the LLO-*KG* strain was consistently, significantly reduced by inhibition of complex V and complex I/III of the ETC, suggesting that oxygen consumption is both directed at ATP production (complex V) and at superoxide formation (I/III).^143^ Interestingly, a compensatory increase in ECAR was observed after complex I/III inhibition, consistent with their interdependence.^142^ Moreover, elevated ECAR with complex I/III inhibition could indicate a role for STAT-1 and KLF6 in inflammation, as complex I generates nicotinamide adenine dinucleotide (NADH) when in reverse electron flow for lactate dehydrogenase to utilize in converting pyruvate to lactate, which contributes to ECAR through H^+^ production.^144,145^ d-Mannose paralleled this result because d-mannose contributes to lactate production in macrophages.^99^ The reduction in OCR, ECAR, and ATP production with a significant shift toward glycolysis (except in LLO-*KG* condition) at 24 h in the d-mannose treatments could have resulted from the suppression of glucose utilization^99^ (Supporting Information Figure S8, Table S2). TAMs have been shown to enhance tumor progression through metabolic changes^145–147^ and rely on glycolysis for energy production, so therapeutic measures have been proposed to increase PPARγ induction to cause increased phagocytic activity, which the LLO-*KG* caused in this study.^145–147^ However, TAM metabolism is understudied and complex, so further studies are needed to understand the ideal target to adjust TAM metabolism.^145–147^ Both the LLO-*SK* and LLO-*KG* strains caused functional changes that could be beneficial in TME altering applications with the LLO-*SK* strain expected to be most efficacious.

The limited examples of efficacy of bacteria immunotherapies when treating late-stage cancers have been related primarily to using pathogenic chassis organisms such as due to direct impact on cancer cell viability.^148,149^ However, when progressing to nonpathogenic chassis organisms and strategies involving activation of the immune system, late-stage cancers (e.g., 4T1 models) have proven challenging to treat.^52,150,151^ The TF strains especially LLO-*SK* are an advance in the use of nonpathogenic chassis organisms that activate the immune system for translatable cancer treatment with efficacy and safety. Initially, the nonpathogenic LLO-*lux* strain was used to visualize location of bacterial accumulation temporally and was observed to be cleared from healthy mice within 24 h, which was also observed with no CFU (Supporting Information Figure S10). However, the bacteria persisted within the tumors for a week postinjection IV and were found to be associated with phagocytes, which established the potential for the engineered LLO strains to be utilized to alter the TME (Supporting Information Figure S9). This potential established by the natural targeting to or accumulation of in the tumor after just an IV injection could have been enhanced by the unique intracellular mechanism and leveraging the transcriptional control of LLO to promote escape from phagosomes only after accumulation in the tumor. Ultimately, the LLO-*SK* caused the greatest inhibition of tumor growth, which was observed at 3 days after initial injection and continued throughout the experiment (Figure 5). LLO-*SK* had improved efficacy compared to LLO-*KG*, which was expected based on the design of the TF pairs and the in vitro results with the BMDMs. LLO-*SK* was injected both IV and IT to reduce tumor progression potentially further as suggested in previous studies.^53,54,69,101^ However, we observed that the dual injections did not improve outcomes relative to single IV injections of LLO-*SK,* which may be explained by immunophenotyping (Figure 5).

Tumor immunophenotyping by flow cytometry provides important insights into the composition of immune cell populations and can be used as a measure of efficacy in therapies, which are designed to promote immune cell invasion into the TME.^106,152,153^ This method has been used in bacterial cancer treatment to understand the benefits of using bacteria to activate immune cells toward treating cancer.^52^ Analysis of the CD45+ hematopoietic cell composition in the 4T1 tumors revealed that the CD11b+ myeloid cell population was the predominant population (average 63.58%) among all CD45+ cells, as seen previously in the 4T1 tumor model.^154^ CD11b+ cells can act in an immunosuppressive manner, inhibiting cancer immunity initiated by T and NK cells.^155^ A reduction in the immunosuppressive CD11b+ and MDSC populations could indicate a therapeutic effect. Although we did not observe a clear change in CD11b+ populations among all the treatment groups, there were reductions in M-MDSC by the LLO-*SK* multiple treatment and LLO +mannose groups (Figure 6). Mannose alone also decreased the M-MDSC population, highlighting how it can contribute to the alteration of the TME and tumor regression.^156,157^ Another CD11b+ population within the TME is TAMs. Commonly, TAMs are of the protumoral, immunosuppressive phenotype (M2), acting to help promote tumor growth and metastases. The predominance of M2 macrophages is associated with a poor outcome, while M1-like macrophages are generally antitumoral.^13,158^ Although we did not observe an increase in the proinflammatory population of TAMs, we could identify a decrease in the anti-inflammatory population in the LLO-*SK* multiple injection and LLO +mannose groups (Figure 6).

T cells are another important immune cell population, which could provide therapeutic benefits during tumor treatment and the LLO*-SK* +mannose did increase the total population of T cells. The total population of CD3+ T cells can be composed of the therapeutic CD4+ or CD8+ populations.^155,159^ On the other hand, the CD4+ population can harbor Tregs (classically defined as CD25+FoxP3+), and in a tumor setting, these cells can suppress the immune response resulting in an inhibition of the ensuing antitumoral immune response.^159,160^ Tregs are often identified using CD25 as an activation marker^161^ and more recently, FoxP3, which is required for development of Tregs and indicates a highly immunosuppressive population.^162^ Our results suggest that mice that were treated with LLO-*SK* +mannose had a decreased population of mature Tregs (CD4+CD25+FoxP3+). Multiple treatments of LLO-*SK* decreased classical precursor Tregs (CD25+FoxP3-), which could have contributed to the limitation of tumor growth in these groups (Figure 6, Supporting Information Figure S12).^163,164^

Tumor metabolism analysis was performed to further understand the processes in the immune modulation goal of the EES platform. Even in the complex TME, we observed an indication toward activated T cell metabolism. Among conditions that had increased T cell populations (CD3+) including tumors within individual groups and across groups, an increase in OCR, ECAR, and ATP production was measured (Supporting Information Figure S13). Activated CD4+ and CD8+ T cells are known to increase both oxidative phosphorylation and glycolysis resulting in increased ATP production during a proinflammatory phenotype indicating various treatments especially LLO-*SK* +mannose were able to increase activated T cells in the TME.^165^ Although the LLO-*SK* multiple injection treatment group had one of the best therapeutic effects, so did the group, which was also injected with LLO-*SK* +mannose but only via two IV doses. Examining immunophenotyping, one may expect the group with multiple injections to have the most promising therapeutic effect. However, it is important to note that the multiple injections seemed to promote changes in macrophage populations, while the single injection stimulated beneficial changes in T cells. Further studies could be performed to examine causes for the immune population differences between a single injection and multiple injection method.

After characterizing negative health effects on the mice, only mice, which received mannose or bacteria +mannose, had a significant reduction in weight except for LLO-*SK* (Supporting Information Figure S11). However, on average, mice that received the treatment had a weight reduction of 2.3%, while the mannose treatment alone reduced weight by 8% indicating mannose in drinking water was the major contributor to weight reduction. Additionally, liver histopathology did not identify any differences between mice bearing 4T1 tumors with no treatment, mannose only treatment, and the bacterial treatments (LLO, LLO-*SK*, LLO-*KG*) with mannose indicating safety of the treatments even at the primary location of initial bacterial accumulation and clearing. This observation is further supported by the clearing of from these organs in healthy mice (Supporting Information Figure S10). The safety observed in the treatments, which is at least comparable if not an improvement over Nissle studies (health of the mice was monitored by body mass and histopathology), increases the translational potential of the work, though the intracellular nature of the EES could be a regulatory concern.

Future directions could include various treatment regimens being tested to potentially improve efficacy, and this could be extended to other cancer models, which would improve translational potential for the EES strategy. For future advancement of the EES in cancer therapy and other applications, an extensive library of TF pairings, beyond these two pairs, should be developed along with improved genetic regulation elements to optimize the macrophage response and improve stability. Additionally, could be designed to target nonphagocytic cells with a protein such as internalin A, which could promote improved results in the TME. The design of bacterial operons expressing mammalian TFs could be improved by eliminating tandem repeats, while also refining control of expressed TF with the aim of maintaining EES stability especially in long-term in vivo experiments.

## Conclusions

Overall, the EES technology has utility for manipulating mammalian immune cell function in a targeted way, showing efficacy and safety as a cancer therapy. As such, the applications of EES could be extended to other biomedical applications that require alteration of mammalian cell function.

## Methods

###  LLO Constructs

 expressing IPTG-inducible LLO was provided by Dr. Daniel Portnoy. LLO *Stat-1Klf6* (LLO-*SK*) and LLO *Klf4Gata-3* (LLO-*KG*) were constructed and utilized in our previous work.^8^ These same strains were utilized in this study and the LLO *luxA-E* (LLO-*luxA-E*) strain was constructed using the same homologous recombination plasmid (pDR111,^166^ a gift from Dr. Lee Kroos) to insert the *luxA-E* operon into the *amyE* locus using a natural competence protocol.^167^ The construct was selected by spectinomycin and then confirmed by PCR amplification out of the genome and bioluminescence imaging. The *luxA-E* operon was amplified from a transposon plasmid used for bioluminescent imaging^105^ and then inserted in place of the *lacI* gene in the pDR111 plasmid using inverse PCR then Gibson cloning for constitutive expression from the Phyper-spank promoter. Accordingly, this construct was inserted in the LLO *amyE* locus (LLO-*luxA-E* or LLO-*lux*).

### Growth Conditions for LLO, LLO-*luxA-E*, and TF Strains

 strains were grown under the same conditions for all of the experiments. Each LLO construct was grown in Luria–Bertani Miller broth (LB) with the appropriate antibiotic. LLO was grown in LB with chloramphenicol (10 μg/mL) while LLO-*SK*, *KG*, and *luxA-E* were grown with spectinomycin (100 μg/mL). The overnight cultures were grown for 16 h at 37 °C and 250 rpm. The LLO-*SK*, *KG*, and *luxA-E* strains were added to macrophages in vitro or injected in vivo without antibiotics because constructs were integrated into the genome.

###  LLO, LLO-*SK*, and LLO-*KG* Addition to BMDMs In Vitro

The following conditions were utilized to induce LLO, LLO-*SK*, and LLO-*KG* delivery, unless otherwise described. BMDMs were sourced from male and female C57BL/6J mice (Jackson Laboratories) of 3–4 months based on previous established methods^9^ and maintained at 37 °C and 5% CO_2_ in DMEM (ThermoFisher, MA, USA), supplemented with 10% fetal bovine serum (FBS), 1% penicillin-streptomycin, and 100 U/mL recombinant macrophage colony-stimulating factor (M-CSF). BMDMs were maintained in these conditions for 7 days before being seeded at 5 × 10^4^ into 96-well plates or 7 × 10^5^-1 × 10^6^ in 6-well plates and allowed to adhere overnight without penicillin–streptomycin. The engineered LLO was added at an optimized MOI of 50:1 for all experiments besides the uptake rate experiment (described below), along with IPTG (500 μM) to induce expression of LLO with or without delivery of TFs. The bacterial strains and BMDMs were then coincubated at 37 °C and 5% CO_2_ for 1 h. BMDMs were then washed three times with PBS, and a new medium was added containing gentamicin (5 μM) to eliminate any remaining extracellular bacteria. Coincubation continued until BMDMs were evaluated by various analyses at various time points with the BMDMs eliminating intracellular engineered LLO within 11 additional hours (Supporting Information Figure 1). Further details of TF delivery in specific experiments are described below.

### Live Cell Imaging

 LLO was added to BMDMs as described above, using a 96-well black glass-bottom plate (50,000 cells/well; PerkinElmer, cat# 6005430). LLO was centrifuged (10,000*g*) for 2 min, then resuspended in CellTracker Orange CMRA Dye (CTO, Invitrogen, C34564, 2 μM) in PBS, and then incubated at 37 °C and 250 rpm for 25 min. Afterward, LLO was centrifuged (10,000*g*) and washed three times before adding to BMDMs. Live cell imaging was performed on a Leica Dmi8 Thunder microscope equipped with a DFC9000 GTC sCMOS camera and LAS-X software (Leica, Wetzlar, Germany). BMDMs were maintained at 37 °C and 5% CO_2_ in a Fluorobrite medium during the imaging session. Fluorescent images of CTO were acquired by using a TRITC filter set. Brightfield and fluorescent images were acquired consecutively, using a 63× oil objective every 1.5 h starting at 3 h postbacterial addition until 9 h postbacterial addition. Z-stacks were taken at all time points at 0.4 μm steps to confirm the presence within the cytoplasm. Zoomed in images were created using Fiji (ImageJ; version 1.53t) software.

### Confirming LLO Phagosomal Escape and Destruction by LC3 Mechanisms

CTO was used to confirm location of LLO after being added to BMDMs in 96-well black glass-bottom plate (50,000 cells/well; PerkinElmer, cat. no. 6005430) as described above. After fixation with 4% paraformaldehyde (PFA), cells were permeabilized using 0.3% Triton X-100 (ThermoFisher) followed by a blocking step containing 0.3% Triton X-100 and 5% normal goat serum (ThermoFisher, cat#31872,). Primary antibodies were incubated at 4 °C overnight followed by secondary antibodies incubated at room temperature (RT) for 2 h. Phagosome formation or destruction was shown by incubating an anti-Lamp-1 ^90^ primary antibody (1:100, AbCam, MA, USA, cat#ab25245) followed by a goat antirat IgG Alexa Fluor 647 secondary antibody (1:5000, ThermoFisher, cat#A-21247). Autophagy mechanisms triggered by LC3 were elucidated by incubating an anti-LC3B primary antibody (1:1000, AbCam, MA, USA, cat#ab192890) followed by a goat antirabbit IgG Texas Red secondary antibody (1:2000, ThermoFisher, cat#T-2767). Nuclei were counterstained by incubating cells with Hoechst 33342 (1 μg/mL) for 10 min (min) at RT. Plates were imaged using a Leica Dmi8 Thunder microscope equipped with a DFC9000 GTC sCMOS camera and LAS-X software (Leica, Wetzlar, Germany). Brightfield and fluorescent images were acquired using a 63× oil objective with the fluorescent images acquired by the DAPI (Hoechst 33342), TRITC (CTO), Texas Red (LC3B), and Cy5 (LAMP-1) filter sets using bandpass filters set on the camera to mitigate fluorescent overlap and imaged in reverse order (Cy5 back toward DAPI) to mitigate exciting the other fluorophores. Overlayed images were created by using Fiji software.

### BMDM Viability and Uptake of LLO by Flow Cytometry

Flow cytometry was used to test for uptake of LLO and change in BMDM viability after bacterial delivery. For viability, conditions examined were multiple time points of interaction between LLO and host cells (4 and 12 h), a 50:1 MOI and with or without IPTG induction compared to untreated with biological triplicates (*n* = 3) for each time and condition. At least 5,000 events were collected from the live gate for analysis. For uptake, conditions examined were multiple MOIs (25:1, 50:1, and 100:1) compared to untreated after 4 h incubation with biological triplicate (*n* = 3) for untreated and 50:1 MOI and one biological replicate (*n* = 1) for 25:1 and 100:1 MOI. Cells were collected, washed once with 1× PBS, and incubated with Zombie NIR viability dye (1:750, Biolegend, San Diego, CA, USA; cat. no. 423105) in PBS for 20 min, at 4 °C in the dark. Cells were washed twice followed by fixation using 4% PFA and resuspended in 100 μL of flow buffer (0.5% bovine serum albumin (BSA), 1× PBS) for analysis using the Cytek Aurora Cytometer (Cytek Biosciences, CA, USA). All samples were assessed for percent live cells. BMDMs, which were incubated with CTO bacteria, were assessed for percent CTO positive cells (BMDMs containing bacteria), based on an FMO cutoff of 0.1% for CTO. Standard one-way ANOVA with Tukey posthoc test was used to determine statistically different values.

### Engineered LLO Strain TF Delivery and BMDM Protein Production Modulation

The LLO strain, LLO-*SK*, and LLO-*KG* were internalized into BMDMs as described above ( LLO, LLO-*SK*, and LLO-*KG* addition to BMDMs in vitro), using a 6-well plate (Corning Costar #3516). D-mannose (1% w/v) was added to controls and to induce TF delivery after the initial 1 h coincubation between BMDMs and bacteria. TFs were delivered throughout the survival of the LLO-*SK* and LLO-*KG* strains and trafficked until experiments were ended for analysis (12, 24, or 48 h). For flow cytometry, Accutase (Sigma, cat#A6964) with scraping was used to detach BMDMs for analysis (described below). For Luminex cytokine/chemokine profiling (Millipore Sigma, MA, USA), the supernatant was removed at both 24 and 48 h, and then, analysis was performed to quantify cytokines/chemokines produced (described below). BMDMs that were untreated were at the resting state. BMDMs were polarized with IFN-γ and LPS (proinflammatory) at 50 and 100 ng/mL, respectively, or IL-4 and IL-13 (anti-inflammatory) at 20 ng/mL each to be used as positive controls. All bacterial strains were treated with and without d-mannose and with IPTG as described above. All treatment conditions were performed in biological triplicates (*n* = 3).

### In Vitro Flow Cytometry

After addition of the engineered LLO strains and controls, followed by incubation for 24 or 48 h, BMDMs were collected and stained in a 96-well round-bottom plate. All staining steps were performed in 100 μL volume at 4 °C in the dark. Samples were first incubated with Zombie NIR viability dye (1:750, Biolegend) for 20 min. Cells were washed once with flow buffer, followed by incubation with TruStain FcX PLUS (antimouse CD16/32) antibody (Biolegend, cat#156603; 0.25 μg/sample) for 10 min. Alexa Fluor 647 antimouse CD86 antibody (0.125 μg/sample; Biolegend; cat#105020) and FITC antimouse CD206 (MMR) antibody (0.1 μg/sample, Biolegend; cat#141703) were then added and incubated for 20 min. Cells were washed twice with flow-staining buffer and fixed with 4% PFA for 10 min and resuspended in a final volume of 100 μL for flow cytometry analysis using the Cytek Aurora spectral flow cytometer (Cytek). Five-thousand events were collected from the live gate for analysis with single-stained controls and unstained controls for all conditions used to assess fluorescent spread and for gating strategies. FMO gates had a cutoff percentage of 0.1%. CD86 and CD206 expression were analyzed from CD11b+/F4/80+ cells. At least 5000 events were collected from the live gate for analysis. Flow cytometry data was analyzed with the software FCSExpress (DeNovo Software, CA, USA). Normality was tested using the Shapiro–Wilk test, and when failed, a Kruskal–Wallis test with Dunn’s multiple comparisons was performed. When normality was identified, a standard one-way ANOVA with Tukey’s multiple comparisons test was used. Statistical tests were used to determine statistically different MFI values among all groups within each time point. The data presented herein were obtained using instrumentation in the MSU Flow Cytometry Core Facility. The facility is funded in part through the financial support of Michigan State University’s Office of Research & Innovation, College of Osteopathic Medicine, and College of Human Medicine.

### Luminex Cytokine/Chemokine Profiling Assay

The cell culture supernatant was stored at −20 °C until use (2 weeks). The supernatant was analyzed for CCL2 (MCP-1), CCL3 (MIP-1a), CXCL2/MIP-2, G-CSF, GM-CSF IL-1β, IL-6, IL-10, IL-12p40, IL-15, TNF-α, and VEGFα cytokine expression. Cytokine and chemokine levels of cell supernatants were measured using a MCYTOMAG-70K Mouse Cytokine Magnetic Multiplex Assay (Millipore Sigma) using a Luminex 200 analyzer instrument (Luminex Corp, USA) according to the manufacturer’s instructions. Standard one-way ANOVA with the Tukey posthoc test was used to determine statistically different values among all treatment groups.

### In Vitro Seahorse Functional Metabolism Assays

Engineered LLO strains were added to BMDMs in a 96-well plate as described above (Engineered *B. subtilis* LLO Strain TF Delivery and BMDM Protein Production Modulation). LPS (100 ng/mL) and D-mannose (1% w/v) served as controls in this experiment. Basal measurements of OCR and ECAR were obtained in real-time using the Seahorse XFe-96 Extracellular Flux Analyzer (Agilent Technologies) and were normalized to cell number.^23,29,168^ Prior to running the assay, the cell culture medium was replaced with the Seahorse XF DMEM medium (pH 7.4) supplemented with 25 mM d-glucose and 4 mM glutamine. The Seahorse ATP rate and cell energy phenotype assays were run according to manufacturer’s instruction, and all reagents for the Seahorse assays were sourced from Agilent Technologies. Wave software (version 2.6.1) was used to process and export Seahorse data. Standard one-way ANOVA with the Tukey posthoc test was used to determine statistically different values among all treatment groups.

### RNA Sequencing

Engineered LLO strains were added to BMDMs in a 96-well plate as described above (Engineered *B. subtilis* LLO Strain TF Delivery and BMDM Protein Production Modulation). The following treatments were used at concentrations described above in biological triplicate at both 12 and 24 h (*n* = 3): untreated, d-mannose, LPS, LPS, and IFN-γ (pro-inflammatory), IL-4 and IL-13 (anti-inflammatory), LLO without IPTG (does not escape phagosomes), LLO strain with and without mannose (LLO -mannose, LLO +mannose), LLO-*SK* with and without mannose (LLO-*SK* -mannose, LLO-*SK* +mannose), and LLO-*KG* with and without mannose (LLO-*KG* -mannose, LLO-*KG* +mannose). These conditions and time points totaled 72 samples of mouse total RNA that was extracted by a Qiagen RNeasy kit (Qiagen, cat#74104) with RNase-free DNase Set (Qiagen, cat#79254) for NGS library preparation and sequencing. Libraries were prepared using the Illumina TruSeq Stranded mRNA Library Preparation Kit with IDT for Illumina TruSeq Unique Dual Index adapters following manufacturer’s recommendations. Completed libraries were quality controlled and quantified using a combination of Qubit dsDNA HS and Agilent 4200 TapeStation HS DNA1000 assays. The libraries were pooled in equimolar quantities for multiplexed sequencing. The library pool was loaded onto one lane of a NovaSeq S4 flow cell; sequencing was performed in a 2 × 150 bp paired end format using a NovaSeq 6000 v1.5 300 cycle reagent cartridge. Base calling was done by Illumina Real Time Analysis (RTA) v3.4.4, and output of RTA was demultiplexed and converted to FastQ format with Illumina Bcl2fastq v2.20.0. All samples reached >30 million read counts. The quality of the reads was evaluated using FastQC (v. 0.11.7). All adaptors were trimmed using Trimmomatic (v. 0.39). The reads were aligned to GRCm39 genome using STAR (v. 2.6.0c). Reads were summarized using featureCounts, and differential gene expression analysis was performed using Deseq2 (v.3.17).

### In Vivo 4T1 Tumor Model and Tumor Growth Measurements

Female BALB/c mice (6–8 weeks; Jackson Laboratories USA) were obtained and cared for in accordance with the standards of the Michigan State University Institutional Animal Care and Use Committee. Mice were anesthetized with isoflurane administered at 2% in oxygen followed by an injection of 200,000 4T1 cells (a gift from Dr. Paula Foster, Western University, *n* = 6 per group (*n* = 7 in LLO-*KG* +mannose group); 98% viability, measured using the trypan blue exclusion assay) suspended in 50 μL PBS into the fourth (inguinal) MFP, as previously reported.^169^ Two weeks postcancer cell implantation (pi) engineered LLO strains treatment was initiated. Mice were randomly divided into groups and separated based on strain injected and presence of d-mannose: (1) no treatment, (2) +mannose +IPTG, (3) LLO-*lux* intravenous (IV) -mannose +IPTG, (4) LLO-*lux* IV +mannose +IPTG, (5) LLO-*SK* IV -mannose +IPTG, (6) LLO-*SK* IV +mannose +IPTG, 7) LLO-*KG* IV -mannose +IPTG, (8) LLO-*KG* IV +mannose +IPTG, and (9) LLO-*SK* IV/IT +mannose +IPTG. Bacterial treatments were administered under anesthesia (as above). The engineered LLO strains were washed three times with PBS after overnight growth before resuspending at 1 × 10^8^ in 100 μL of PBS and then injected IV or resuspended and injected in 25 μL of PBS for IT. After 24 h postbacterial treatment, groups, which were to be given d-mannose and IPTG, received an IP injection of each (2 g/kg d-mannose, 50 mg/kg IPTG) and added to water (20% w/v d-mannose, 40 mM IPTG). Seven days after the first bacterial treatment, a second bacterial treatment was given, followed by d-mannose and IPTG 24 h after. After the initial bacterial injection, animal well-being was documented every 2–3 days by observing water consumption, grooming, and weight measurements. Tumors were measured using calipers beginning on the first day of the first treatment and continued every observation day of the second week until end point. Tumor volume was calculated using the equation:^170^ tumor volume = 0.5­(length × width^2^) from measurements taken by at least two individuals. A repeated measures two-way ANOVA with the Tukey posthoc test were used to determine any significance between treatments and time points. At the end point, tumors were collected for flow cytometry immunophenotyping, histology, measurement of metabolism, and colony-forming units (below). Livers were also collected in formalin for histology to determine the damage to tissue from repeated bacterial injections.

### Imaging of LLO-*lux* Strains In Vivo

The LLO-*lux* strain was imaged using the IVIS system (IVIS Spectrum, PerkinElmer) using autoexposure settings (time = 120–300 s, binning = medium, f/stop = 1, emission filter = open). The LLO-*lux* strain was imaged immediately after injection, 1 h postinjection, 24 h postinjection, 72 h postinjection, and at following regular time points that correlate with caliper measurements and animal well-being documentation. A final imaging time point was taken before euthanasia and then after the tumors were removed and cut in half to elucidate the location of bacteria throughout the tumor. Additionally, LLO-*lux* was injected into four mice (*n* = 4) without tumors (-tumor) and imaged to track clearance of the bacteria using methods with tumor-bearing mice mentioned above.

### Tumor Immunophenotyping and Metabolism Characterization

Tumors were collected from each group (*n* = 3) and halved for digestion into single cell suspension followed by immunophenotyping using flow cytometry analysis or metabolism characterization using the Seahorse assay. Tumors were minced mechanically followed by digestion using a solution containing DMEM, collagenase III (300 U/ml; Worthington Biochemical, cat. no. LS004182) and DNase I (100 U/ml; Worthington Biochemical, cat#LS002139). Tumors were digested for 90 min at 37 °C, 5% CO_2_ with agitation using a pipet every 30 min. The solution was passed through a 70 μm strainer followed by centrifugation and resuspension in ACK Lysing Buffer (ThermoFisher, cat#A1049201) for 1 min followed by addition of HBSS + 10% FBS. Cells were centrifuged, resuspended in PBS, and counted using the Trypan Blue assay. For immunophenotyping, 1 × 10^6^ cells (*n* = 2) were collected per sample and transferred into a Nunc MicroWell 96-well polypropylene plate (Millipore Sigma, cat#P6866–1CS) for staining. All staining steps were performed in 100 μL volume at 4 °C in the dark. Samples were first incubated with LIVE/DEAD Fixable Blue Dead Cell Stain (1:500, ThermoFisher, catalog no. L23105) for 30 min. Cells were washed once with flow buffer, followed by incubation with TruStain FcX PLUS (antimouse CD16/32) antibody (Biolegend, cat#156603; 0.25 μg/sample) for 10 min. A mixture of the following antibodies was then added to the samples for 30 min: CD11b PE (0.125 ug/sample; Biolegend, cat#101207), CD8a BUV737 (0.25 μg/sample; ThermoFisher, cat#367–0081–80), CD25 SuperBright 780 (0.0625 μg/sample; ThermoFisher, cat#78–0251–82), CD3 APC/Fire 810 (0.25 μg/sample; Biolegend, cat#100267), Ly-6C PE/Cyanine7 (1:200; Biolegend, cat#128017), Ly-6G Alexa Fluor 700 (1:200; Biolegend, cat#127621), CD4 Brilliant Violet 510 (1:300; Biolegend, cat#100449), MHC-II (I-A/I-E) Spark Blue 550 (0.25 μg/sample; Biolegend, cat#107661), CD206 BV421 (1.25 μL/sample; Biolegend, cat#141717), NKp46 BV605 (1:100; Biolegend, cat#137619), CD11c BB700 (1:200; BD Bioscience, cat#566505), CD45 BUV395 (1:400; BD Bioscience, cat#564279), and CD19 BUV615 (0.125 ug/sample; BD Bioscience, cat#751213). Cells were washed followed by fixation and permeabilization for intracellular staining as per manufacturer’s protocol (eBioscience, cat#00–5523–00). FOXP3 AF647 (2.5 μL/sample; Biolegend, cat. no. 320014) was then added to the samples for 30 min. Cells were washed twice with permeabilization buffer and resuspended in a final volume of 100 μL for flow cytometry analysis using a Cytek Aurora spectral flow cytometer (Cytek). Single stained controls and unstained controls for all conditions were used to assess fluorescent spread, and FMO controls were used for gating strategies when needed. FMO gates had a cutoff percentage of 0.1%. At least 5000 events were collected from the live gate for analysis. Flow cytometry data was analyzed with the software FCSExpress (DeNovo Software). For immunophenotyping, normality was tested using the Shapiro–Wilk test, and when it failed, a Kruskal–Wallis test with Dunn’s multiple comparisons was performed. When normality was identified, a standard one-way ANOVA with Tukey’s multiple comparisons test was used. Statistical tests were used to compare all treatments.

For Seahorse analysis, 5 × 10^4^ cells from the halved and dissociated tumors in immunophenotyping above (*n* = 3) were seeded into an appropriate 96-well plate in technical replicates of three wells (*n* = 3). The Seahorse Mito Stress assay was run according to the manufacturer’s instruction, and all reagents for the Seahorse assays were sourced from Agilent Technologies. For the Seahorse analysis, a Brown-Forsythe and Welch ANOVA with Dunnett T3 posthoc test was used to determine statistically different values among all treatment groups. For the association analysis between immunophenotyping and metabolism, a linear regression model was used for statistical analysis.

### Colony-Forming Units of LLO Strains In Vivo

After tumors were halved for immunophenotyping metabolism, two of the three alternate halves were used for CFU. Additionally, two whole tumors from the remaining three mice in the groups and half of the final tumor were used. Whole tumors or halves were weighed and then homogenized in 1 mL of PBS using a 1.5 mm zirconium bead tube (Benchmark, cat#D1032–15) by bead beating in a Benchmark Beadbug 6 Microtube Homogenizer at max speed for 10 min. One-hundred microliters of the homogenized mixture was then spread on LB plates with spectinomycin (100 μg/mL) for all groups, and a 1:100 dilution was included for the LLO-*SK* IV+IT group. CFUs were counted, and concentrations in tumors were calculated based on dilutions and then normalized to tumor mass (g). The same process was repeated using whole livers and spleens from the three mice (*n* = 3) used for immunophenotyping to calculate CFU in those organs including an additional three mice (*n* = 3) from the tumor group. Brown–Forsythe and Welch ANOVA with the Dunnett T3 posthoc test was used to determine any statistically different values among the treatment groups.

### Histological Analysis

After the final time point, tumors and livers were collected and fixed in 4% PFA for 24 h. Tumors underwent cryopreservation through serial submersion in sucrose (10, 20, and 30%). Tumors were frozen in optimal cutting temperature compound (Fisher HealthCare, USA) and sectioned using a cryostat (4 μm sections) and placed on a slide for staining. Sections were submerged in deionized water for 5 min followed by blocking with 2% BSA in 1× PBS for 30 min. Sections were then incubated with the anti-CD11b polyclonal antibody (ThermoFisher, cat#PA5–79532, 1 ug/mL) for 2 h at room temperature. Sections were washed twice with 1× PBS followed by incubation with a secondary antirabbit IgG, HRP-linked antibody (Cell Signaling Technology, cat#7074S, 1:2000) for 30 min. Sections were washed twice with 1× PBS followed by chromogenic detection using a DAB substrate kit (ThermoFisher, cat. no. 34002) as per manufacturers suggestion. Sections were then stained to identify bacteria using a Gram Stain Kit (ThermoFisher, cat#R40080) and the following procedure: incubate with crystal violet (5 min), rinse with DI water, incubate with Gram’s iodine (2 min), and decolorize using 95% ethanol (30 s). Sections were then dehydrated using 95 and 100% ethanol, followed by clearing using xylene. Sections were imaged using a Nikon Eclipse Ci microscope equipped with a Nikon DS-Fi3 camera (Nikon, Tokyo, Japan) for color acquisition and NIS elements’ BR 5.21.02 software.

Formalin-fixed samples of liver were sent to the MSU Veterinary Diagnostic Laboratory to be analyzed by a board-certified veterinary pathologist (Dr. Matti Kiupel). Briefly, samples were paraffin embedded after being processed in a Leica PELORIS II Premium Tissue Processing System using a standard 8 h xylene-free isopropanol schedule followed by sectioning (3 μm) and routine hematoxylin and eosin (H&E) and Giemsa staining.

### Statistical Analysis and Reproducibility

Statistical analyses were performed using Prism software (9.4.0, GraphPad Inc., La Jolla, CA). Statistical tests are identified for each method. Data are expressed as mean ± standard deviation unless stated otherwise; *p* <.05 was considered a significant finding. Plotting was performed using R version 4.0.4 with the following packages: ggplot2, dplyr, reshape2, ggsignif, plotrix, and ggpubr.

## Supplementary Material



## Data Availability

All raw data and engineered LLO constructs will be made available upon request by the corresponding author. Raw data used in the RNA-seq analysis can be accessed at Gene Expression Omnibus (GEO #GSE239519).

## Notes

EES design and modulation of the tumor microenvironment, method to deliver EES to BMDMs, live cell imaging of replicating EES in live BMDMs, genome-wide gene expression shifts in EES-treated BMDMs, uptake efficiency and viability of BMDM after EES uptake, changes in surface markers on BMDMs following treatment with EES, profiling of BMDM cytokine and chemokine production following treatment with EES, patterns of functional metabolism for BMDMs following treatment with EES, quantification of functional metabolism shifts at 12 h, quantification of functional metabolism shifts at 24 h, in vivo delivery of EES, colony-forming units from tumors and organs, body weight and organ weight, summary of liver histopathology, immunophenotyping of tumors following treatment with EES, and functional metabolism of cells in tumor following treatment with EES (PDF)

## Notes

All R scripts were written with a general format appropriate for the openly available, established packages mentioned above, and can be made available on request. Access to 3D visualizations and code for RNA-seq analysis are available on GitHub (https://github.com/madsen16/Engineered-endosymbionts).

## §

Department of Imaging Physics, University of Texas MD Anderson, 1515 Holcombe Boulevard, Houston, Texas 77030, United States

## #

BioFrontiers Institute, University of Colorado, 3415 Colorado Avenue, Boulder, Colorado 80303, United States

## References

[1] Elston K. M., Leonard S. P., Geng P., Bialik S. B., Robinson E., Barrick J. E. (2022). Engineering Insects from the Endosymbiont Out. Trends Microbiol.

[2] Jiggins F. M. (2017). The Spread
of Wolbachia through Mosquito Populations. PLoS
Biol..

[3] Flores H. A., O’Neill S. L. (2018). Controlling
Vector-Borne Diseases by Releasing Modified
Mosquitoes. Nat. Rev. Microbiol.

[4] Leonard S. P., Powell J. E., Perutka J., Geng P., Heckmann L. C., Horak R. D., Davies B. W., Ellington A. D., Barrick J. E., Moran N. A. (2020). Engineered Symbionts
Activate Honey
Bee Immunity and Limit Pathogens. Science.

[5] Machado R. A. R., Thönen L., Arce C. C. M., Theepan V., Prada F., Wüthrich D., Robert C. A. M., Vogiatzaki E., Shi Y. M., Schaeren O. P., Notter M., Bruggmann R., Hapfelmeier S., Bode H. B., Erb M. (2020). Engineering Bacterial
Symbionts of Nematodes Improves Their Biocontrol Potential to Counter
the Western Corn Rootworm. Nat. Biotechnol..

[6] Shi X.-B., Yan S., Zhang C., Zheng L.-M., Zhang Z.-H., Sun S.-E., Gao Y., Tan X.-Q., Zhang D.-Y., Zhou X.-G. (2021). Aphid Endosymbiont
Facilitates Virus Transmission by Modulating the Volatile Profile
of Host Plants. BMC Plant Biol..

[7] Kennedy J., Marchesi J. R., Dobson A. D. W. (2007). Metagenomic
Approaches to Exploit
the Biotechnological Potential of the Microbial Consortia of Marine
Sponges. Appl. Microbiol. Biotechnol..

[8] Madsen C. S., Makela A. V., Greeson E. M., Hardy J. W., Contag C. H. (2022). Engineered
Endosymbionts That Alter Mammalian Cell Surface Marker, Cytokine and
Chemokine Expression. Commun. Biol..

[9] Gonçalves R., Mosser D. M. (2015). The Isolation and
Characterization of Murine Macrophages. Curr.
Protoc. Immunol..

[10] Zhang X., Goncalves R., Mosser D. M. (2008). The Isolation and
Characterization
of Murine Macrophages. Curr. Protoc. Immunol..

[11] Germic N., Frangez Z., Yousefi S., Simon H.-U. (2019). Regulation of the
Innate Immune System by Autophagy: Monocytes, Macrophages, Dendritic
Cells and Antigen Presentation. Cell Death Differ..

[12] Frucht D. M., Fukao T., Bogdan C., Schindler H., O’Shea J. J., Koyasu S. (2001). IFN-γ Production by Antigen-Presenting
Cells: Mechanisms Emerge. Trends Immunol.

[13] Mosser D. M., Edwards J. P. (2008). Exploring the Full Spectrum of Macrophage Activation. Nat. Rev. Immunol..

[14] Lavin Y., Winter D., Blecher-Gonen R., David E., Keren-Shaul H., Merad M., Jung S., Amit I. (2014). Tissue-Resident Macrophage
Enhancer Landscapes Are Shaped by the Local Microenvironment. Cell.

[15] Mantovani A., Sica A., Sozzani S., Allavena P., Vecchi A., Locati M. (2004). The Chemokine System in Diverse Forms
of Macrophage
Activation and Polarization. Trends Immunol.

[16] Das A., Yang C.-S., Arifuzzaman S., Kim S., Kim S. Y., Jung K. H., Lee Y. S., Chai Y. G. (2018). High-Resolution
Mapping and Dynamics of the Transcriptome, Transcription Factors,
and Transcription Co-Factor Networks in Classically and Alternatively
Activated Macrophages. Front Immunol.

[17] Turner M. D., Nedjai B., Hurst T., Pennington D. J. (2014). Cytokines
and Chemokines: At the Crossroads of Cell Signalling and Inflammatory
Disease. Biochimica et Biophysica Acta (BBA) - Molecular. Cell Research.

[18] Martinez F. O., Sica A., Mantovani A., Locati M. (2008). Macrophage Activation
and Polarization. Front. Biosci..

[19] Liu Y., Xu R., Gu H., Zhang E., Qu J., Cao W., Huang X., Yan H., He J., Cai Z. (2021). Metabolic
Reprogramming in Macrophage Responses. Biomark
Res..

[20] Kelly B., O’neill L. A. (2015). Metabolic
Reprogramming in Macrophages and Dendritic
Cells in Innate Immunity. Cell Res..

[21] Galván-Peña S., O’Neill L. A. J. (2014). Metabolic
Reprograming in Macrophage Polarization. Front.
Immunol..

[22] Blagih J., Jones R. G. (2012). Polarizing Macrophages through Reprogramming
of Glucose
Metabolism. Cell Metab.

[23] Ip W. K. E., Hoshi N., Shouval D. S., Snapper S., Medzhitov R. (2017). Anti-Inflammatory
Effect of IL-10 Mediated by Metabolic Reprogramming of Macrophages. Science (1979).

[24] Chen Z., Lu W., Garcia-Prieto C., Huang P. (2007). The Warburg Effect
and Its Cancer Therapeutic Implications. J.
Bioenerg Biomembr.

[25] Escoll P., Buchrieser C. (2018). Metabolic Reprogramming of Host Cells
upon Bacterial
Infection: Why Shift to a Warburg-like Metabolism?. FEBS J..

[26] Liu D., Chang C., Lu N., Wang X., Lu Q., Ren X., Ren P., Zhao D., Wang L., Zhu Y., He F., Tang L. (2017). Comprehensive Proteomics Analysis Reveals Metabolic
Reprogramming of Tumor-Associated Macrophages Stimulated by the Tumor
Microenvironment. J. Proteome Res..

[27] Curi R., de Siqueira Mendes R., de Campos Crispin L.
A., Norata G. D., Sampaio S. C., Newsholme P. (2017). A Past and Present Overview of Macrophage
Metabolism and Functional Outcomes. Clin Sci..

[28] Pelletier M., Billingham L. K., Ramaswamy M., Siegel R. M. (2014). Chapter Seven -
Extracellular Flux Analysis to Monitor Glycolytic Rates and Mitochondrial
Oxygen Consumption. Methods Enzymol..

[29] Tannahill G. M., Curtis A. M., Adamik J., Palsson-McDermott E. M., McGettrick A. F., Goel G., Frezza C., Bernard N. J., Kelly B., Foley N. H., Zheng L., Gardet A., Tong Z., Jany S. S., Corr S. C., Haneklaus M., Caffrey B. E., Pierce K., Walmsley S., Beasley F. C., Cummins E., Nizet V., Whyte M., Taylor C. T., Lin H., Masters S. L., Gottlieb E., Kelly V. P., Clish C., Auron P. E., Xavier R. J., O’Neill L. A. J. (2013). Succinate
Is an Inflammatory Signal That Induces IL-1β through HIF-1α. Nature.

[30] Divakaruni A. S., Paradyse A., Ferrick D. A., Murphy A. N., Jastroch M. (2014). Chapter Sixteen
- Analysis and Interpretation of Microplate-Based Oxygen Consumption
and PH Data. Methods Enzymol..

[31] Cho H. J., Jung J. I., Lim D. Y., Kwon G. T., Her S., Park J. H., Park J. H. Y. (2012). Bone
Marrow-Derived, Alternatively
Activated Macrophages Enhance Solid Tumor Growth and Lung Metastasis
of Mammary Carcinoma Cells in a Balb/C Mouse Orthotopic Model. Breast Cancer Res..

[32] Redente E. F., Dwyer-Nield L. D., Merrick D. T., Raina K., Agarwal R., Pao W., Rice P. L., Shroyer K. R., Malkinson A. M. (2010). Tumor Progression
Stage and Anatomical Site Regulate Tumor-Associated Macrophage and
Bone Marrow-Derived Monocyte Polarization. Am.
J. Pathol..

[33] Pathria P., Louis T. L., Varner J. A. (2019). Targeting Tumor-Associated
Macrophages
in Cancer. Trends Immunol.

[34] Davies L. C., Rosas M., Jenkins S. J., Liao C.-T., Scurr M. J., Brombacher F., Fraser D. J., Allen J. E., Jones S. A., Taylor P. R. (2013). Distinct
Bone Marrow-Derived and Tissue-Resident Macrophage
Lineages Proliferate at Key Stages during Inflammation. Nat. Commun..

[35] Redente E.
F., Higgins D. M., Dwyer-Nield L. D., Orme I. M., Gonzalez-Juarrero M., Malkinson A. M. (2010). Differential Polarization of Alveolar Macrophages and
Bone Marrow-Derived Monocytes Following Chemically and Pathogen-Induced
Chronic Lung Inflammation. J. Leukoc Biol..

[36] Xia Y., Rao L., Yao H., Wang Z., Ning P., Chen X. (2020). Engineering
Macrophages for Cancer Immunotherapy and Drug Delivery. Adv. Mater..

[37] Spiller K. L., Koh T. J. (2017). Macrophage-Based
Therapeutic Strategies in Regenerative
Medicine. Adv. Drug Deliv Rev..

[38] Kuhn M., Goebel W. (1994). Induction of Cytokines
in Phagocytic Mammalian Cells
Infected with Virulent and Avirulent Listeria Strains. Infect. Immun..

[39] Fortier A., Faucher S. P., Diallo K., Gros P. (2011). Global Cellular Changes
Induced by Legionella Pneumophila Infection of Bone Marrow-Derived
Macrophages. Immunobiology.

[40] Das A., Sinha M., Datta S., Abas M., Chaffee S., Sen C. K., Roy S. (2015). Monocyte and
Macrophage Plasticity
in Tissue Repair and Regeneration. Am. J. Pathol..

[41] Puurunen M. K., Vockley J., Searle S. L., Sacharow S. J., Phillips J. A., Denney W. S., Goodlett B. D., Wagner D. A., Blankstein L., Castillo M. J., Charbonneau M. R., Isabella V. M., Sethuraman V. V., Riese R. J., Kurtz C. B., Brennan A. M. (2021). Safety and Pharmacodynamics
of an Engineered E. Coli Nissle for the Treatment of Phenylketonuria:
A First-in-Human Phase 1/2a Study. Nat. Metab.

[42] Charbonneau M. R., Denney W. S., Horvath N. G., Cantarella P., Castillo M. J., Puurunen M. K., Brennan A. M. (2021). Development of a
Mechanistic Model to Predict Synthetic Biotic Activity in Healthy
Volunteers and Patients with Phenylketonuria. Commun. Biol..

[43] Sedighi M., Zahedi Bialvaei A., Hamblin M. R., Ohadi E., Asadi A., Halajzadeh M., Lohrasbi V., Mohammadzadeh N., Amiriani T., Krutova M., Amini A., Kouhsari E. (2019). Therapeutic
Bacteria to Combat Cancer; Current Advances, Challenges, and Opportunities. Cancer Med..

[44] Sawant S. S., Patil S. M., Gupta V., Kunda N. K. (2020). Microbes as Medicines:
Harnessing the Power of Bacteria in Advancing Cancer Treatment. Int. J. Mol. Sci..

[45] Yaghoubi A., Khazaei M., Hasanian S. M., Avan A., Cho W. C., Soleimanpour S. (2019). Bacteriotherapy
in Breast Cancer. Int. J. Mol. Sci..

[46] Soleimanpour S., Hasanian S. M., Avan A., Yaghoubi A., Khazaei M. (2020). Bacteriotherapy
in Gastrointestinal Cancer. Life Sci..

[47] Zhang Z. H., Yin L., Zhang L. L., Song J. (2020). Efficacy and Safety of Bacillus Calmette-Guerin
for Bladder Cancer: A Protocol of Systematic Review. Medicine (Baltimore).

[48] Sfakianos J. P., Salome B., Daza J., Farkas A., Bhardwaj N., Horowitz A. (2021). Bacillus Calmette-Guerin
(BCG): Its Fight against Pathogens
and Cancer. Urol Oncol.

[49] La
Mantia I., Varricchio A., Di Girolamo S., Minni A., Passali G. C., Ciprandi G. (2019). The Role of Bacteriotherapy
in the Prevention of Adenoidectomy. Eur. Rev.
Med. Pharmacol. Sci..

[50] Andaloro C., Santagati M., Stefani S., La Mantia I. (2019). Bacteriotherapy
with Streptococcus Salivarius 24SMB and Streptococcus Oralis 89a Oral
Spray for Children with Recurrent Streptococcal Pharyngotonsillitis:
A Randomized Placebo-Controlled Clinical Study. Eur. Arch Otorhinolaryngol.

[51] Yaghoubi A., Khazaei M., Jalili S., Hasanian S. M., Avan A., Soleimanpour S., Cho W. C. (2020). Bacteria as a Double-Action Sword
in Cancer. Biochim Biophys Acta Rev. Cancer.

[52] Chowdhury S., Castro S., Coker C., Hinchliffe T. E., Arpaia N., Danino T. (2019). Programmable Bacteria Induce Durable
Tumor Regression and Systemic Antitumor Immunity. Nat. Med..

[53] Din M. O., Danino T., Prindle A., Skalak M., Selimkhanov J., Allen K., Julio E., Atolia E., Tsimring L. S., Bhatia S. N., Hasty J. (2016). Synchronized Cycles
of Bacterial
Lysis for in Vivo Delivery. Nature.

[54] Chien T., Harimoto T., Kepecs B., Gray K., Coker C., Hou N., Pu K., Azad T., Nolasco A., Pavlicova M., Danino T. (2021). Enhancing the Tropism of Bacteria via Genetically Programmed
Biosensors. Nat. Biomed. Eng..

[55] Danino T., Lo J., Prindle A., Hasty J., Bhatia S. N. (2012). In Vivo Gene Expression
Dynamics of Tumor-Targeted Bacteria. ACS Synth.
Biol..

[56] Abedi M. H., Yao M. S., Mittelstein D. R., Bar-Zion A., Swift M. B., Lee-Gosselin A., Barturen-Larrea P., Buss M. T., Shapiro M. G. (2022). Ultrasound-Controllable
Engineered Bacteria for Cancer Immunotherapy. Nat. Commun..

[57] He L., Yang H., Tang J., Liu Z., Chen Y., Lu B., He H., Tang S., Sun Y., Liu F., Ding X., Zhang Y., Hu S., Xia L. (2019). Intestinal
Probiotics E. Coli Nissle 1917 as a Targeted Vehicle for Delivery
of P53 and Tum-5 to Solid Tumors for Cancer Therapy. J. Biol. Eng..

[58] Yu X., Lin C., Yu J., Qi Q., Wang Q. (2020). Bioengineered
Escherichia
Coli Nissle 1917 for Tumour-targeting Therapy. Microb Biotechnol.

[59] Danino T., Prindle A., Kwong G. A., Skalak M., Li H., Allen K., Hasty J., Bhatia S. N. (2015). Programmable Probiotics
for Detection of Cancer in Urine. Sci. Transl.
Med..

[60] Zhang Y., Zhang Y., Xia L., Zhang X., Ding X., Yan F., Wu F. (2012). Escherichia
Coli Nissle 1917 Targets and Restrains
Mouse B16 Melanoma and 4T1 Breast Tumors through Expression of Azurin
Protein. Appl. Environ. Microbiol..

[61] Luke J. J., Piha-Paul S. A., Medina T., Verschraegen C. F., Varterasian M., Brennan A. M., Riese R. J., Sokolovska A., Strauss J., Hava D. L., Janku F. (2023). Phase I Study of SYNB1891,
an Engineered E. Coli Nissle Strain Expressing STING Agonist, with
and without Atezolizumab in Advanced Malignancies. Clin. Cancer Res..

[62] Flickinger J. C., Rodeck U., Snook A. E. (2018). Listeria
Monocytogenes as a Vector
for Cancer Immunotherapy: Current Understanding and Progress. Vaccines.

[63] Morrow Z. T., Powers Z. M., Sauer J.-D. (2019). Listeria
Monocytogenes Cancer Vaccines:
Bridging Innate and Adaptive Immunity. Curr.
Clin Microbiol Rep.

[64] Hayashi K., Zhao M., Yamauchi K., Yamamoto N., Tsuchiya H., Tomita K., Hoffman R. M. (2009). Cancer Metastasis
Directly Eradicated
by Targeted Therapy with a Modified Salmonella Typhimurium. J. Cell Biochem.

[65] Nagakura C., Hayashi K., Zhao M., Yamauchi K., Yamamoto N., Tsuchiya H., Tomita K., Bouvet M., Hoffman R. M. (2009). Efficacy
of a Genetically-Modified Salmonella Typhimurium in an Orthotopic
Human Pancreatic Cancer in Nude Mice. Anticancer
Res..

[66] Liang K., Liu Q., Li P., Luo H., Wang H., Kong Q. (2019). Genetically
Engineered Salmonella Typhimurium: Recent Advances in Cancer Therapy. Cancer Lett..

[67] Nguyen V. H., Kim H.-S., Ha J.-M., Hong Y., Choy H. E., Min J.-J. (2010). Genetically Engineered Salmonella
Typhimurium as an
Imageable Therapeutic Probe for Cancer. Cancer
Res..

[68] Guo Y., Chen Y., Liu X., Min J.-J., Tan W., Zheng J. H. (2020). Targeted Cancer Immunotherapy with Genetically Engineered
Oncolytic Salmonella Typhimurium. Cancer Lett..

[69] Harimoto T., Hahn J., Chen Y.-Y., Im J., Zhang J., Hou N., Li F., Coker C., Gray K., Harr N., Chowdhury S., Pu K., Nimura C., Arpaia N., Leong K. W., Danino T. (2022). A Programmable
Encapsulation System
Improves Delivery of Therapeutic Bacteria in Mice. Nat. Biotechnol..

[70] Toso J. F., Gill V. J., Hwu P., Marincola F. M., Restifo N. P., Schwartzentruber D. J., Sherry R. M., Topalian S. L., Yang J. C., Stock F., Freezer L. J., Morton K. E., Seipp C., Haworth L., Mavroukakis S., White D., MacDonald S., Mao J., Sznol M., Rosenberg S. A. (2002). Phase I Study of the Intravenous Administration of
Attenuated Salmonella Typhimurium to Patients with Metastatic Melanoma. J. Clin Oncol.

[71] Yelin I., Flett K. B., Merakou C., Mehrotra P., Stam J., Snesrud E., Hinkle M., Lesho E., McGann P., McAdam A. J., Sandora T. J., Kishony R., Priebe G. P. (2019). Genomic
and Epidemiological Evidence of Bacterial Transmission from Probiotic
Capsule to Blood in ICU Patients. Nat. Med..

[72] Bielecki J., Youngman P., Connelly P., Portnoy D. A. (1990). Bacillus Subtilis
Expressing a Haemolysin Gene from Listeria Monocytogenes Can. Grow
in Mammalian Cells. Nature.

[73] Zuber P., Losick R. (1987). Role of AbrB in Spo0A-
and Spo0B-Dependent Utilization
of a Sporulation Promoter in Bacillus Subtilis. J. Bacteriol..

[74] Travassos L. H., Girardin S. E., Philpott D. J., Blanot D., Nahori M.-A., Werts C., Boneca I. G. (2004). Toll-like
Receptor 2-Dependent Bacterial
Sensing Does Not Occur via Peptidoglycan Recognition. EMBO Rep.

[75] Elshaghabee F. M. F., Rokana N., Gulhane R. D., Sharma C., Panwar H. (2017). Bacillus As
Potential Probiotics: Status, Concerns, and Future Perspectives. Front Microbiol.

[76] Kolkman M. A. B., Van Der Ploeg R., Bertels M., Van Dijk M., Van Der Laan J., Van Dijl J. M., Ferrari E. (2008). The Twin-Arginine Signal
Peptide of Bacillus Subtilis YwbN Can Direct Either Tat- or Sec-Dependent
Secretion of Different Cargo Proteins: Secretion of Active Subtilisin
via the B. Subtilis Tat Pathway. Appl. Environ.
Microbiol.

[77] Mitchell P. J., Tjian R. (1989). Transcriptional Regulation in Mammalian Cells by Sequence-Specific
DNA Binding Proteins. Science.

[78] Fleetwood A. J., Lawrence T., Hamilton J. A., Cook A. D. (2007). Granulocyte-Macrophage
Colony-Stimulating Factor (CSF) and Macrophage CSF-Dependent Macrophage
Phenotypes Display Differences in Cytokine Profiles and Transcription
Factor Activities: Implications for CSF Blockade in Inflammation. J. Immunol..

[79] Heinz S., Benner C., Spann N., Bertolino E., Lin Y. C., Laslo P., Cheng J. X., Murre C., Singh H., Glass C. K. (2010). Simple Combinations
of Lineage-Determining
Transcription Factors Prime Cis-Regulatory Elements Required for Macrophage
and B Cell Identities. Mol. Cell.

[80] Tugal D., Liao X., Jain M. K. (2013). Transcriptional
Control of Macrophage
Polarization. Arterioscler., Thromb., Vasc.
Biol..

[81] Rilo-Alvarez H., Ledo A. M., Vidal A., Garcia-Fuentes M. (2021). Delivery of
Transcription Factors as Modulators of Cell Differentiation. Drug Deliv Transl Res..

[82] Li H., Jiang T., Li M.-Q., Zheng X.-L., Zhao G.-J. (2018). Transcriptional
Regulation of Macrophages Polarization by MicroRNAs. Front. Immunol..

[83] Yang M., Song L., Wang L., Yukht A., Ruther H., Li F., Qin M., Ghiasi H., Sharifi B. G., Shah P. K. (2018). Deficiency
of GATA3-Positive Macrophages Improves Cardiac Function Following
Myocardial Infarction or Pressure Overload Hypertrophy. J. Am. Coll. Cardiol..

[84] VanDeusen J. B., Shah M. H., Becknell B., Blaser B. W., Ferketich A. K., Nuovo G. J., Ahmer B. M. M., Durbin J., Caligiuri M. A. (2006). STAT-1-Mediated
Repression of Monocyte Interleukin-10 Gene Expression in Vivo. Eur. J. Immunol..

[85] Date D., Das R., Narla G., Simon D. I., Jain M. K., Mahabeleshwar G. H. (2014). Kruppel-like
Transcription Factor 6 Regulates Inflammatory Macrophage Polarization. J. Biol. Chem..

[86] Liao X., Sharma N., Kapadia F., Zhou G., Lu Y., Hong H., Paruchuri K., Mahabeleshwar G. H., Dalmas E., Venteclef N., Flask C. A., Kim J., Doreian B. W., Lu K. Q., Kaestner K. H., Hamik A., Clément K., Jain M. K. (2011). Krüppel-like Factor 4 Regulates
Macrophage Polarization. J. Clin. Invest..

[87] Tao K., Fang M., Alroy J., Sahagian G. G. (2008). Imagable 4T1Model
for the Study of Late Stage Breast Cancer. BMC
Cancer.

[88] Zhang H., Xie W., Zhang Y., Dong X., Liu C., Yi J., Zhang S., Wen C., Zheng L., Wang H. (2022). Oncolytic
Adenoviruses Synergistically Enhance Anti-PD-L1 and Anti-CTLA-4 Immunotherapy
by Modulating the Tumour Microenvironment in a 4T1 Orthotopic Mouse
Model. Cancer Gene Ther..

[89] Madsen, C. S. Advancing Engineered Endosymbionts as a Platform Technology for Therapeutic Macrophage Modulation; PhD Dissertation. Michigan State University, 2022

[90] Kortebi M., Milohanic E., Mitchell G., Péchoux C., Prevost M.-C., Cossart P., Bierne H. (2017). Listeria Monocytogenes
Switches from Dissemination to Persistence by Adopting a Vacuolar
Lifestyle in Epithelial Cells. PLoS Pathog.

[91] Huynh K. K., Eskelinen E.-L., Scott C. C., Malevanets A., Saftig P., Grinstein S. (2007). LAMP Proteins Are Required for Fusion
of Lysosomes with Phagosomes. EMBO J..

[92] Deretic V., Saitoh T., Akira S. (2013). Autophagy in Infection, Inflammation
and Immunity. Nat. Rev. Immunol.

[93] Byrne B. G., Dubuisson J. F., Joshi A. D., Persson J. J., Swanson M. S., Zychlinsky A. (2013). Inflammasome
Components Coordinate Autophagy and Pyroptosis
as Macrophage Responses to Infection. mBio.

[94] Levine B., Mizushima N., Virgin H. W. (2011). Autophagy in Immunity
and Inflammation. Nature.

[95] Siqueira M. da S., Ribeiro R. de M., Travassos L. H. (2018). Autophagy
and Its Interaction With Intracellular Bacterial Pathogens. Front Immunol.

[96] Gordon S., Martinez F. O. (2010). Alternative Activation
of Macrophages: Mechanism and
Functions. Immunity.

[97] Orecchioni M., Ghosheh Y., Pramod A. B., Ley K. (2019). Macrophage Polarization:
Different Gene Signatures in M1­(LPS+) vs. Classically and M2­(LPS−)
vs. Alternatively Activated Macrophages. Front
Immunol.

[98] Viola A., Munari F., Sánchez-Rodríguez R., Scolaro T., Castegna A. (2019). The Metabolic Signature of Macrophage
Responses. Front Immunol.

[99] Torretta S., Scagliola A., Ricci L., Mainini F., Di Marco S., Cuccovillo I., Kajaste-Rudnitski A., Sumpton D., Ryan K. M., Cardaci S. (2020). D-Mannose Suppresses
Macrophage IL-1β Production. Nat. Commun..

[100] LeBleu V. S., O’Connell J. T., Gonzalez Herrera K. N., Wikman H., Pantel K., Haigis M. C., de Carvalho F. M., Damascena A., Domingos Chinen L. T., Rocha R. M., Asara J. M., Kalluri R. (2014). PGC-1α Mediates
Mitochondrial Biogenesis and
Oxidative Phosphorylation in Cancer Cells to Promote Metastasis. Nat. Cell Biol..

[101] Hong E.-H., Chang S.-Y., Lee B.-R., Pyun A.-R., Kim J.-W., Kweon M.-N., Ko H.-J. (2013). Intratumoral
Injection
of Attenuated Salmonella Vaccine Can Induce Tumor Microenvironmental
Shift from Immune Suppressive to Immunogenic. Vaccine.

[102] Duong M. T.-Q., Qin Y., You S.-H., Min J.-J. (2019). Bacteria-Cancer
Interactions: Bacteria-Based Cancer Therapy. Exp Mol. Med..

[103] Sieow B. F.-L., Wun K. S., Yong W. P., Hwang I. Y., Chang M. W. (2021). Tweak to Treat: Reprograming Bacteria for Cancer Treatment. Trends Cancer.

[104] Francis K. P., Joh D., Bellinger-Kawahara C., Hawkinson M. J., Purchio T. F., Contag P. R. (2000). Monitoring Bioluminescent
Staphylococcus Aureus Infections in Living Mice Using a Novel LuxABCDE
Construct. Infect. Immun..

[105] Francis K. P., Yu J., Bellinger-Kawahara C., Joh D., Hawkinson M. J., Xiao G., Purchio T. F., Caparon M. G., Lipsitch M., Contag P. R. (2001). Visualizing Pneumococcal Infections
in the Lungs of Live Mice Using Bioluminescent Streptococcus Pneumoniae
Transformed with a Novel Gram-Positive Lux Transposon. Infect. Immun..

[106] Pockley A. G., Foulds G. A., Oughton J. A., Kerkvliet N. I., Multhoff G. (2015). Immune Cell Phenotyping Using Flow Cytometry. Curr. Protoc. Toxicol..

[107] Sun T., Altenbuchner J. (2010). Characterization
of a Mannose Utilization System in
Bacillus Subtilis. J. Bacteriol..

[108] Schindler C., Levy D. E., Decker T. (2007). JAK-STAT Signaling:
From Interferons to Cytokines. J. Biol. Chem..

[109] Yagi R., Zhu J., Paul W. E. (2011). An Updated View
on Transcription Factor GATA3-Mediated Regulation of Th1 and Th2 Cell
Differentiation. Int. Immunol..

[110] Ryu Y. H., Baik J. E., Yang J. S., Kang S. S., Im J., Yun C. H., Kim D. W., Lee K., Chung D. K., Ju H. R., Han S. H. (2009). Differential Immunostimulatory
Effects
of Gram-Positive Bacteria Due to Their Lipoteichoic Acids. Int. Immunopharmacol..

[111] Moreira L. O., El Kasmi K. C., Smith A. M., Finkelstein D., Fillon S., Kim Y., Núñez G., Tuomanen E., Murray P. J. (2008). The TLR2-MyD88-NOD2-RIPK2 Signalling
Axis Regulates a Balanced Pro-inflammatory and IL-10-mediated Anti-inflammatory
Cytokine Response to Gram-positive Cell Walls. Cell Microbiol.

[112] Holden J. A., Attard T. J., Laughton K. M., Mansell A., O’Brien-Simpson N. M., Reynolds E. C. (2014). Porphyromonas
Gingivalis
Lipopolysaccharide Weakly Activates M1 and M2 Polarized Mouse Macrophages
but Induces Inflammatory Cytokines. Infect.
Immun..

[113] Jaynes J. M., Sable R., Ronzetti M., Bautista W., Knotts Z., Abisoye-Ogunniyan A., Li D., Calvo R., Dashnyam M., Singh A., Guerin T., White J., Ravichandran S., Kumar P., Talsania K., Chen V., Ghebremedhin A., Karanam B., Bin Salam A., Amin R., Odzorig T., Aiken T., Nguyen V., Bian Y., Zarif J. C., de Groot A. E., Mehta M., Fan L., Hu X., Simeonov A., Pate N., Abu-Asab M., Ferrer M., Southall N., Ock C. Y., Zhao Y., Lopez H., Kozlov S., de Val N., Yates C. C., Baljinnyam B., Marugan J., Rudloff U. (2020). Mannose Receptor (CD206)
Activation in Tumor-Associated Macrophages Enhances Adaptive and Innate
Antitumor Immune Responses. Sci. Transl. Med..

[114] García-González G., Sánchez-González A., Hernández-Bello R., González G. M., Franco-Molina M. A., Coronado-Cerda E. E., Palma-Nicolás J. P. (2019). Triggering
of Protease-Activated Receptors (PARs) Induces Alternative M2Macrophage
Polarization with Impaired Plasticity. Mol.
Immunol.

[115] Leber N., Kaps L., Yang A., Aslam M., Giardino M., Klefenz A., Choteschovsky N., Rosigkeit S., Mostafa A., Nuhn L., Schuppan D., Zentel R. (2019). α-Mannosyl-Functionalized
Cationic Nanohydrogel
Particles for Targeted Gene Knockdown in Immunosuppressive Macrophages. Macromol. Biosci.

[116] Stahl P. D., Ezekowitz R. A. B. (1998). The
Mannose Receptor Is a Pattern
Recognition Receptor Involved in Host Defense. Curr. Opin Immunol.

[117] Murtaugh M. P., Foss D. L. (2002). Inflammatory Cytokines and Antigen
Presenting Cell Activation. Vet. Immunol. Immunopathol..

[118] Zhang X., Mosser D. M. (2008). Macrophage Activation by Endogenous
Danger Signals. J. Pathol..

[119] Wang L., Zhang S., Wu H., Rong X., Guo J. (2019). M2b Macrophage
Polarization and Its Roles in Diseases. J. Leukoc
Biol..

[120] Graves D. T. (1999). The Potential Role of Chemokines and Inflammatory Cytokines
in Periodontal Disease Progression. Clinical
Infectious Diseases.

[121] Tanaka T., Bai Z., Srinoulprasert Y., Yang B., Hayasaka H., Miyasaka M. (2005). Chemokines in Tumor
Progression and Metastasis. Cancer Sci..

[122] Ben-Baruch A. (2002). Host Microenvironment
in Breast Cancer Development:
Inflammatory Cells, Cytokines and Chemokines in Breast Cancer Progression:
Reciprocal Tumor-Microenvironment Interactions. Breast Cancer Res..

[123] Raman D., Baugher P. J., Thu Y. M., Richmond A. (2007). Role of Chemokines
in Tumor Growth. Cancer Lett..

[124] Benoit M., Desnues B., Mege J.-L. (2008). Macrophage Polarization
in Bacterial Infections. J. Immunol..

[125] Smith T. D., Tse M. J., Read E. L., Liu W. F. (2016). Regulation
of Macrophage Polarization and Plasticity by Complex Activation Signals. Integr Biol. (Camb).

[126] Rőszer T. (2015). Understanding the Mysterious M2Macrophage
through Activation
Markers and Effector Mechanisms. Mediators Inflamm.

[127] Martins A., Han J., Kim S. O. (2010). The Multifaceted
Effects of Granulocyte Colony-Stimulating Factor in Immunomodulation
and Potential Roles in Intestinal Immune Homeostasis. IUBMB Life.

[128] Becker C., Wirtz S., Ma X., Blessing M., Galle P. R., Neurath M. F. (2001). Regulation of IL-12 p40 Promoter
Activity in Primary Human Monocytes: Roles of NF-κB, CCAAT/Enhancer-Binding
Protein β, and PU.1 and Identification of a Novel Repressor
Element (GA-12) That Responds to IL-4 and Prostaglandin E2. J. Immunol..

[129] Laha D., Grant R., Mishra P., Nilubol N. (2021). The Role of
Tumor Necrosis Factor in Manipulating the Immunological Response of
Tumor Microenvironment. Front Immunol.

[130] Rosenzweig J. M., Glenn J. D., Calabresi P. A., Whartenby K. A. (2013). KLF4Modulates Expression of IL-6 in Dendritic Cells
via Both Promoter Activation and Epigenetic Modification. J. Biol. Chem..

[131] Deshmane S. L., Kremlev S., Amini S., Sawaya B. E. (2009). Monocyte
Chemoattractant Protein-1 (MCP-1): An Overview. J. Interferon Cytokine Res..

[132] Sutcliffe A. M., Clarke D. L., Bradbury D. A., Corbett L. M., Patel J. A., Knox A. J. (2009). Transcriptional
Regulation of Monocyte
Chemotactic Protein-1 Release by Endothelin-1 in Human Airway Smooth
Muscle Cells Involves NF-KappaB and AP-1. Br.
J. Pharmacol..

[133] Kanemaru H., Yamane F., Fukushima K., Matsuki T., Kawasaki T., Ebina I., Kuniyoshi K., Tanaka H., Maruyama K., Maeda K., Satoh T., Akira S. (2017). Antitumor effect of Batf2 through IL-12 p40 up-regulation in tumor-associated
macrophages. Proc. Natl. Acad. Sci. U. S. A..

[134] Kenny P. A., Lee G. Y., Bissell M. J. (2007). Targeting
the Tumor
Microenvironment. Front Biosci.

[135] Chonov D. C., Ignatova M. M. K., Ananiev J. R., Gulubova M. V. (2019). IL-6 Activities
in the Tumour Microenvironment. Part 1. Open Access Maced. J. Med. Sci..

[136] Hu J., Zhao Q., Kong L.-Y., Wang J., Yan J., Xia X., Jia Z., Heimberger A. B., Li S. (2021). Regulation of Tumor
Immune Suppression and Cancer Cell Survival by CXCL1/2 Elevation in
Glioblastoma Multiforme. Sci. Adv..

[137] Burke S. J., Lu D., Sparer T. E., Masi T., Goff M. R., Karlstad M. D., Collier J. J. (2014). NF-ΚB and
STAT1 Control CXCL1 and CXCL2 Gene Transcription. Am. J. Physiol Endocrinol Metab.

[138] Laoui D., Movahedi K., Van Overmeire E., Van den Bossche J., Schouppe E., Mommer C., Nikolaou A., Morias Y., De Baetselier P., Van Ginderachter J. A. (2011). Tumor-Associated
Macrophages in Breast Cancer: Distinct Subsets, Distinct Functions. Int. J. Dev Biol..

[139] Bhavsar I., Miller C. S., Al-Sabbagh M., Preedy V. R., Patel V. B. (2015). Macrophage Inflammatory Protein-1
Alpha (MIP-1 Alpha)/CCL3: As a Biomarker. Gen.
Methods Biomarker Res. Appl..

[140] Errea A., Cayet D., Marchetti P., Tang C., Kluza J., Offermanns S., Sirard J.-C., Rumbo M. (2016). Lactate Inhibits the Pro-Inflammatory
Response and Metabolic Reprogramming in Murine Macrophages in a GPR81-Independent
Manner. PLoS One.

[141] Maduka C. V., Habeeb O. M., Kuhnert M. M., Hakun M., Goodman S. B., Contag C. H. (2023). Glycolytic Reprogramming
Underlies
Immune Cell Activation by Polyethylene Wear Particles. Biomaterials Advances.

[142] van Raam B. J., Sluiter W., de Wit E., Roos D., Verhoeven A. J., Kuijpers T. W., Khoury J. E. (2008). Mitochondrial Membrane
Potential in Human Neutrophils Is Maintained by Complex III Activity
in the Absence of Supercomplex Organisation. PLoS One.

[143] Maduka C. V., Kuhnert M. M., Habeeb O. M., Tundo A., Makela A. V., Goodman S. B., Contag C. H. (2023). Elevated
Oxidative
Phosphorylation Is Critical for Immune Cell Activation by Polyethylene
Wear Particles. J. Immunol Regen Med..

[144] Mookerjee S. A., Gerencser A. A., Nicholls D. G., Brand M. D. (2017). Quantifying
Intracellular Rates of Glycolytic and Oxidative ATP Production and
Consumption Using Extracellular Flux Measurements. J. Biol. Chem..

[145] Diskin C., Pålsson-McDermott E. M. (2018). Metabolic
Modulation
in Macrophage Effector Function. Front Immunol.

[146] Rabold K., Netea M. G., Adema G. J., Netea-Maier R. T. (2017). Cellular
Metabolism of Tumor-associated Macrophages-Functional Impact and Consequences. FEBS Lett..

[147] Wculek S. K., Dunphy G., Heras-Murillo I., Mastrangelo A., Sancho D. (2022). Metabolism of Tissue Macrophages
in Homeostasis and Pathology. Cell Mol. Immunol.

[148] Jawalagatti V., Kirthika P., Lee J. H. (2022). Targeting Primary
and Metastatic Tumor Growth in an Aggressive Breast Cancer by Engineered
Tryptophan Auxotrophic Salmonella Typhimurium. Mol. Ther Oncolytics.

[149] Geng Z., Cao Z., Liu R., Liu K., Liu J., Tan W. (2021). Aptamer-Assisted
Tumor Localization of Bacteria for
Enhanced Biotherapy. Nat. Commun..

[150] Pan H., Li L., Pang G., Han C., Liu B., Zhang Y., Shen Y., Sun T., Liu J., Chang J., Wang H. (2021). Engineered NIR Light-Responsive Bacteria
as Anti-Tumor Agent for Targeted and Precise Cancer Therapy. Chemical Engineering Journal.

[151] Zheng D.-W., Chen Y., Li Z.-H., Xu L., Li C.-X., Li B., Fan J.-X., Cheng S.-X., Zhang X.-Z. (2018). Optically-Controlled Bacterial Metabolite for Cancer
Therapy. Nat. Commun..

[152] Daud A. I., Loo K., Pauli M. L., Sanchez-Rodriguez R., Sandoval P. M., Taravati K., Tsai K., Nosrati A., Nardo L., Alvarado M. D., Algazi A. P., Pampaloni M. H., Lobach I. V., Hwang J., Pierce R. H., Gratz I. K., Krummel M. F., Rosenblum M. D. (2016). Tumor Immune
Profiling Predicts Response
to Anti–PD-1 Therapy in Human Melanoma. J. Clin. Invest..

[153] Khalsa J. K., Cheng N., Keegan J., Chaudry A., Driver J., Bi W. L., Lederer J., Shah K. (2020). Immune Phenotyping
of Diverse Syngeneic Murine Brain Tumors Identifies Immunologically
Distinct Types. Nat. Commun..

[154] DuPré S. A., Redelman D., Hunter K. W. J. (2007). The
Mouse Mammary
Carcinoma 4T1: Characterization of the Cellular Landscape of Primary
Tumours and Metastatic Tumour Foci. Int. J.
Exp Pathol.

[155] Bronte V., Brandau S., Chen S.-H., Colombo M. P., Frey A. B., Greten T. F., Mandruzzato S., Murray P. J., Ochoa A., Ostrand-Rosenberg S., Rodriguez P. C., Sica A., Umansky V., Vonderheide R. H., Gabrilovich D. I. (2016). Recommendations for Myeloid-Derived Suppressor Cell
Nomenclature and Characterization Standards. Nat. Commun..

[156] Gonzalez P. S., O’Prey J., Cardaci S., Barthet V. J. A., Sakamaki J. i., Beaumatin F., Roseweir A., Gay D. M., Mackay G., Malviya G., Kania E., Ritchie S., Baudot A. D., Zunino B., Mrowinska A., Nixon C., Ennis D., Hoyle A., Millan D., McNeish I. A., Sansom O. J., Edwards J., Ryan K. M. (2018). Mannose
Impairs Tumour Growth and Enhances Chemotherapy. Nature.

[157] Liu Q., Li X., Zhang H., Li H. (2022). Mannose Attenuates
Colitis-Associated Colorectal Tumorigenesis by Targeting Tumor-Associated
Macrophages. J. Cancer Prev.

[158] Moreira A. P., Hogaboam C. M. (2011). Macrophages in Allergic
Asthma: Fine-Tuning
Their pro- and Anti-Inflammatory Actions for Disease Resolution. J. Interferon Cytokine Res..

[159] Rech A. J., Mick R., Martin S., Recio A., Aqui N. A., Powell D. J., Colligon T. A., Trosko J. A., Leinbach L. I., Pletcher C. H., Tweed C. K., DeMichele A., Fox K. R., Domchek S. M., Riley J. L., Vonderheide R. H. (2012). CD25 Blockade
Depletes and Selectively Reprograms Regulatory T Cells in Concert
with Immunotherapy in Cancer Patients. Sci.
Transl. Med..

[160] Sharma S., Yang S.-C., Zhu L., Reckamp K., Gardner B., Baratelli F., Huang M., Batra R. K., Dubinett S. M. (2005). Tumor Cyclooxygenase-2/Prostaglandin
E2–Dependent
Promotion of FOXP3 Expression and CD4+ CD25+ T Regulatory Cell Activities
in Lung Cancer. Cancer Res..

[161] Lopez-Cabrera M., Santis A. G., Fernandez-Ruiz E., Blacher R., Esch F., Sanchez-Mateos P., Sanchez-Madrid F. (1993). Molecular Cloning, Expression, and Chromosomal Localization
of the Human Earliest Lymphocyte Activation Antigen AIM/CD69, a New
Member of the C-Type Animal Lectin Superfamily of Signal-Transmitting
Receptors. J. Exp Med..

[162] Fontenot J. D., Gavin M. A., Rudensky A. Y. (2003). Foxp3 Programs
the
Development and Function of CD4+ CD25+ Regulatory T Cells. Nat. Immunol.

[163] Lio C.-W. J., Hsieh C.-S. (2008). A Two-Step Process for Thymic Regulatory
T Cell Development. Immunity.

[164] Vang K. B., Yang J., Mahmud S. A., Burchill M. A., Vegoe A. L., Farrar M. A. (2008). IL-2,-7, and-15,
but Not Thymic Stromal
Lymphopoeitin, Redundantly Govern CD4+ Foxp3+ Regulatory T Cell Development. J. Immunol..

[165] Klein Geltink R. I., Kyle R. L., Pearce E. L. (2018). Unraveling
the Complex
Interplay between T Cell Metabolism and Function. Annu. Rev. Immunol..

[166] Rokop M. E., Auchtung J. M., Grossman A. D. (2004). Control
of DNA Replication
Initiation by Recruitment of an Essential Initiation Protein to the
Membrane of Bacillus Subtilis. Mol. Microbiol..

[167] Harwood, C. R. ; Cutting, S. M. ; Chambert, R. Molecular Biological Methods for Bacillus. Wiley 1990

[168] Mills E.
L., Kelly B., Logan A., Costa A. S. H., Varma M., Bryant C. E., Tourlomousis P., Däbritz J. H.
M., Gottlieb E., Latorre I., Corr S. C., McManus G., Ryan D., Jacobs H. T., Szibor M., Xavier R. J., Braun T., Frezza C., Murphy M. P., O’Neill L. A. (2016). Succinate
Dehydrogenase Supports Metabolic Repurposing
of Mitochondria to Drive Inflammatory Macrophages. Cell.

[169] Zhang G.-L., Zhang Y., Cao K.-X., Wang X.-M. (2019). Orthotopic
Injection of Breast Cancer Cells into the Mice Mammary Fat Pad. JoVE.

[170] Liu X., Pu Y., Cron K., Deng L., Kline J., Frazier W. A., Xu H., Peng H., Fu Y.-X., Xu M. M. (2015). CD47 Blockade Triggers
T Cell-Mediated Destruction of Immunogenic
Tumors. Nat. Med..

