# Supplementary material for: Engineered Endosymbionts that Modulate Primary Macrophage Function and Attenuate Tumor Growth by Shifting the Tumor Microenvironment
Source: ACS Appl Bio Mater. 2025 Jun 24;8(7):5938–58. doi: 10.1021/acsabm.5c00590 (PMC12284892; doi:10.1021/acsabm.5c00590)
Supplement: Supplementary file 1 [file mt5c00590_si_001.pdf]

Supporting Information

Engineered endosymbionts that modulate primary macrophage function and attenuate tumor growth by shifting the tumor microenvironment

Cody S. Madsen[1,2,3##‡], Ashley V. Makela[2,3‡§], Chima V. Maduka[2,4#], Emily M. Greeson[2,5], Anthony Tundo[2,6], Evran Ural[2,3], Satyajit Hari Kulkarni[2,4], Ahmed A. Zarea[2,7], Matti Kiupel[8], Maryam Sayadi[2,3] and Christopher H. Contag[2,3,5*]

[1]Nuclear and Chemical Sciences Division, Lawrence Livermore National Laboratory, 7000 East Avenue, Livermore, CA, 94550 USA
[2]Institute for Quantitative Health Science and Engineering, Michigan State University, 775 Woodlot Drive, East Lansing, MI, 48823 USA
[3]Department of Biomedical Engineering, Michigan State University, 775 Woodlot Drive, East Lansing, MI, 48823 USA
[4]Comparative Medicine & Integrative Biology, Michigan State University, 775 Woodlot Drive, East Lansing, MI, 48823 USA
[5]Department of Microbiology Genetics and Immunology, Michigan State University, 775 Woodlot Drive, East Lansing, MI, 48823 USA
[6]Department of Biochemistry and Molecular Biology, Michigan State University, 775 Woodlot Drive, East Lansing, MI, 48823 USA
[7]Program in Cellular and Molecular Biology, Michigan State University, 775 Woodlot Drive,East Lansing, MI, 48823 USA
[8]Department of Pathobiology and Diagnostic Investigation, Michigan State University, 775 Woodlot Drive, East Lansing, MI, 48823 USA

Current address:
#BioFrontiers Institute, University of Colorado, 3415 Colorado Avenue, Boulder, CO, 80303 USA
§Department of Imaging Physics, University of Texas MD Anderson, 1515 Holcombe Boulevard, Houston TX, 77030 USA

Prior address:
##Institute for Quantitative Health Science and Engineering, and Department of Biomedical Engineering, Michigan State University, 775 Woodlot Drive, East Lansing, MI, USA

‡ CSM and AVM contributed equally to this paper
* corresponding author
Email: contagch@msu.edu

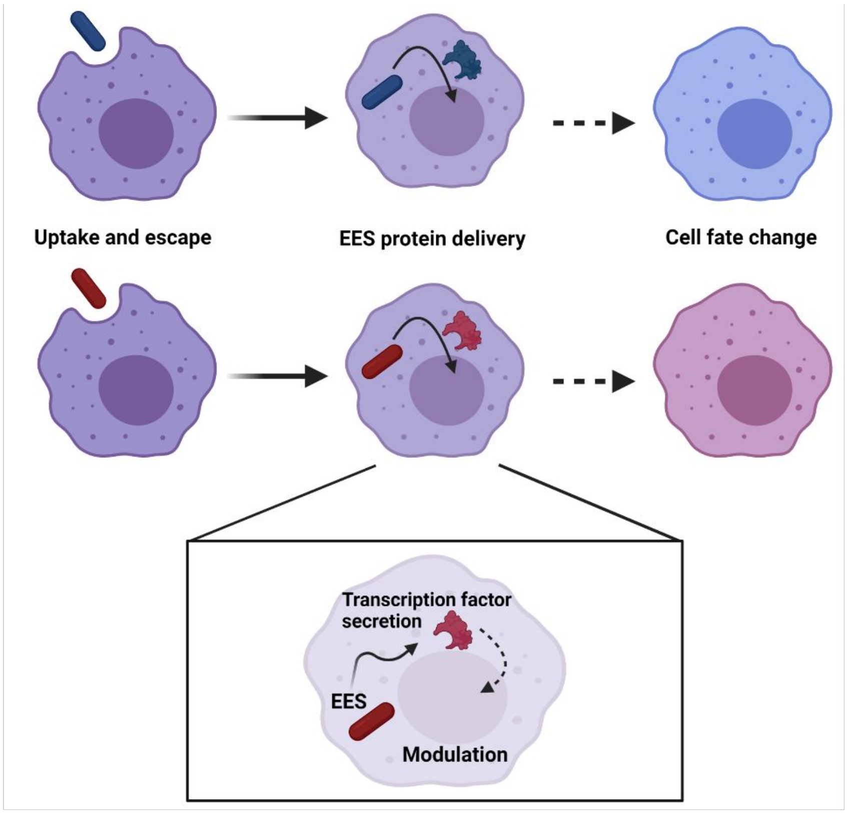
Uptake and escape
EES protein delivery
Cell fate change
Transcription factor secretion
EES
Modulation

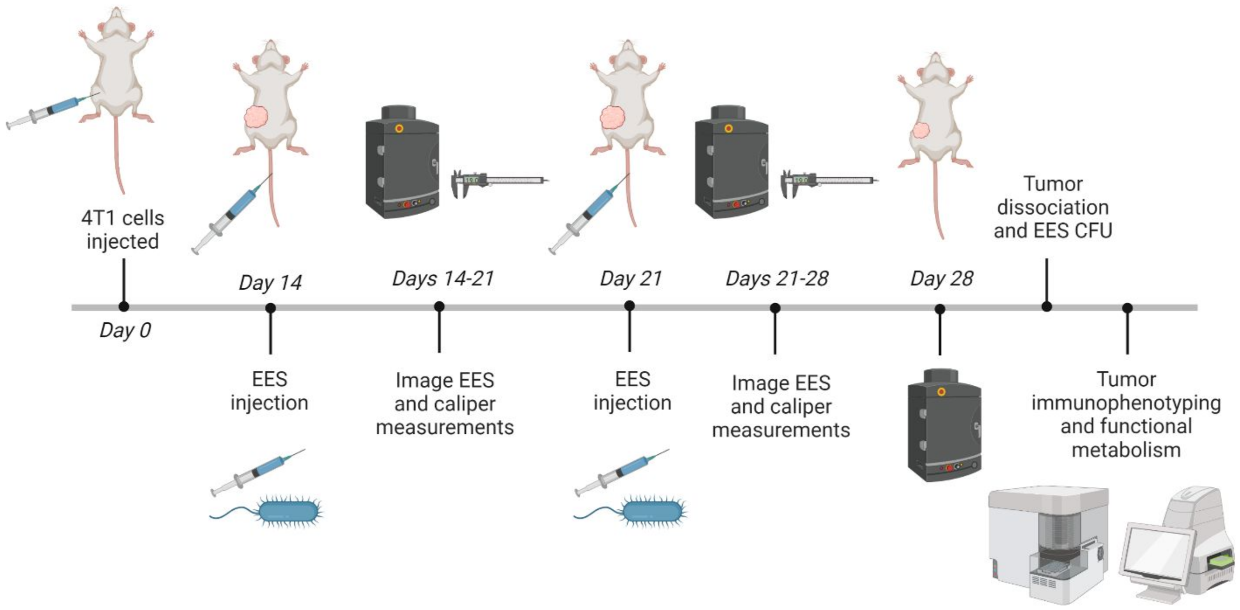
4T1 cells injected
Day 0
Day 14
EES injection
Days 14-21
Image EES and caliper measurements
Day 21
EES injection
Days 21-28
Image EES and caliper measurements
Day 28
Tumor dissociation and EES CFU
Tumor immunophenotyping and functional metabolism

Supplementary Fig. S1. The design of EES to modulate macrophage function and demonstration of EES-mediated modulation of the tumor microenvironment

The EES are taken up by macrophages and escape into the cytoplasm to deliver TFs to modulate host cell function. To demonstrate utility redirecting the TME, we used an orthotopic 4T1 breast cancer model in mice and evaluated the localization of EES and effects on the TME. The EES localization and persistence were tracked using an In Vivo Imaging System (IVIS), and tumor growth was measured by calipers. Tumors were characterized by immunophenotyping (flow cytometry), functional metabolism (Seahorse real-time metabolic assays) and EES colony forming units (CFU).

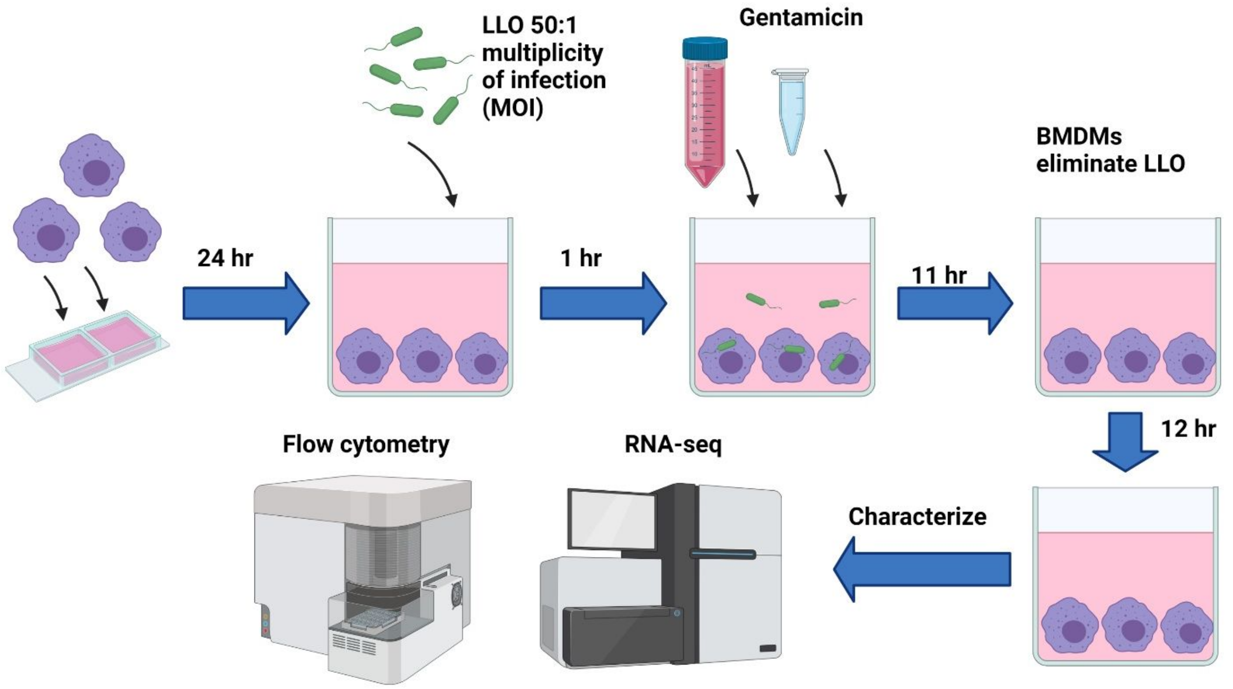

Supplementary Fig. S2. Diagram of method to deliver *B. subtilis* LLO strains to BMDMs and analyze interactions

B. subtilis LLO were co-incubated with host BMDMs according to the timeline shown and analyzed. Bacteria were incubated with BMDMs for 1 h then gentamicin was added to eliminate extracellular bacteria. After 11 additional hours of incubation, BMDMs were observed to eliminate all intracellular bacteria. Analysis was performed at multiple time intervals but in most cases, incubation was continued for an additional 12 h to determine impact on host cells by flow cytometry and RNA-sequencing.

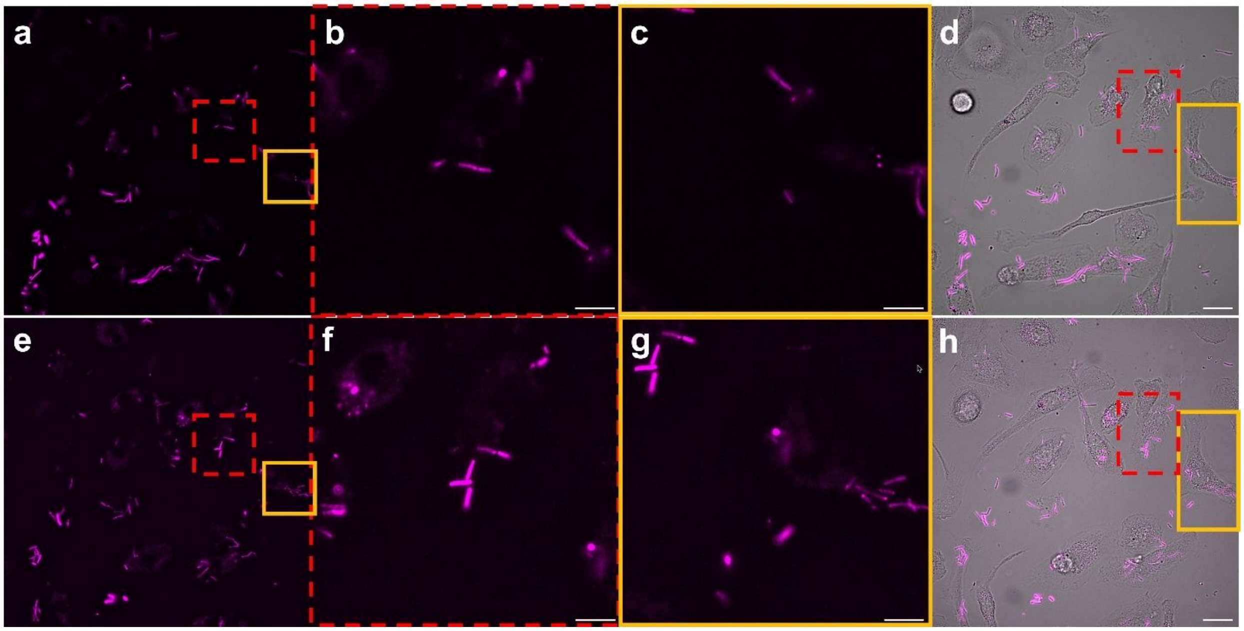

Supplementary Fig. S3. Live cell imaging of replicating *B. subtilis* LLO inside live BMDMs

Live cell microscopy revealed the LLO strain replicating in multiple host cells by comparing images at 3 h (top images, a-d) and 4.5 h (bottom images, e-f) post-bacterial addition. BMDMs were visualized in brightfield (d, h), and the LLO strain using fluorescence (magenta); zoomed images (b-d and f-g) reveal bacteria (magenta LLO-expressing strain of *B. subtilis*) replicating in the cytoplasm and the regions at higher resolution are marked on low resolution images with red dashed box or a yellow solid box. Scale bars = 3 μm for zoomed images (b-c and f-g) and scale bars = 20 μm for not zoomed images are in overlay images (d, h).

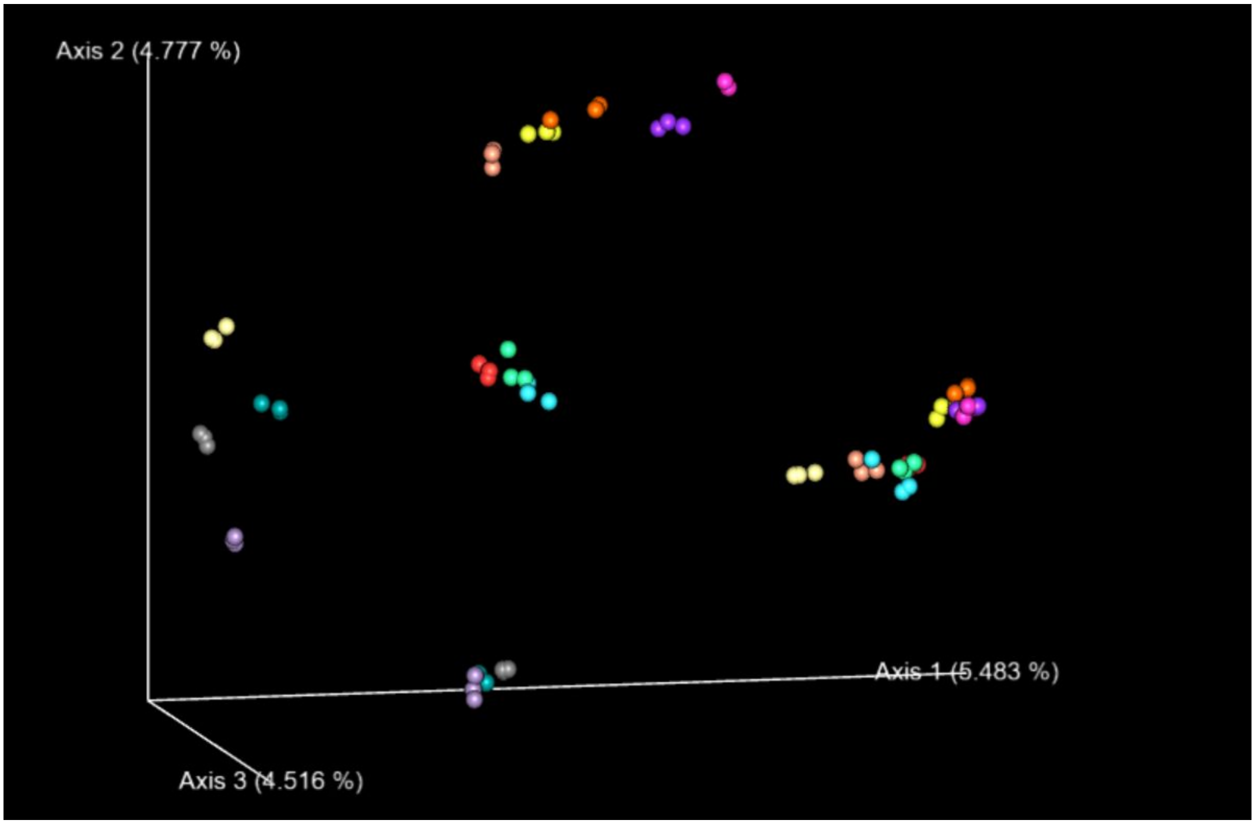

Supplementary Fig. S4. Genome-wide gene expression shifts in BMDMs after response to engineered *B. subtilis* LLO strains and controls

QIIME 2 Emperor Plot visualizes the shifts in genome-wide gene expression after BMDMs were untreated (gray), treated with LPS (salmon), mannose (lavender), LPS and IFN-γ (pink), IL-4 and IL-13 (teal), LLO strain without IPTG (no IPTG, sand), LLO strain with and without mannose (LLO -mannose, orange; LLO +mannose, neon green), LLO-*SK* with and without mannose (LLO-*SK* -mannose, purple; LLO-*SK* +mannose, light blue) and LLO-*KG* with and without mannose (LLO-*KG* -mannose, yellow; LLO-*KG* +mannose, red) at 12 and 24 h post-initial treatment. The 24 h treatments are positioned in the middle, left and upper side of the plot while the 12 h are on the bottom and right side of the plot in this 2D view. The 3D visualization can be accessed through QIIME 2 view by the process explained in the GitHub.

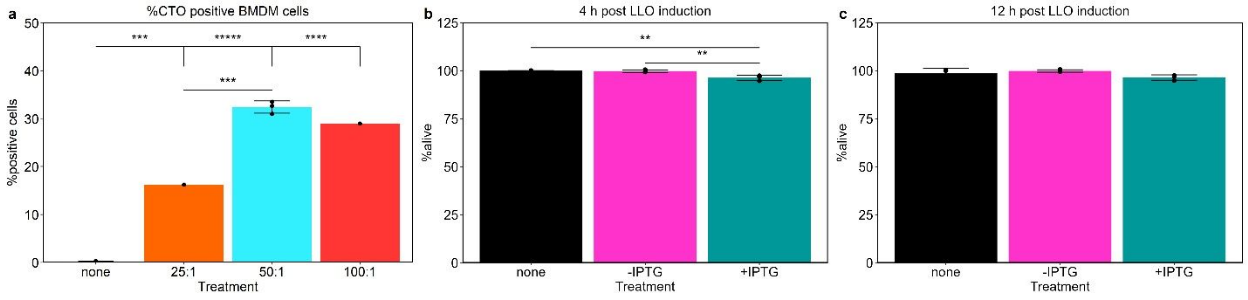

Supplementary Fig. S5. Uptake of *B. subtilis* LLO by BMDM and assessment of BMDM viability after uptake

BMDMs were co-incubated with *B. subtilis* LLO strain (+IPTG) which were stained with CellTracker Orange (CTO) CMRA Dye. CTO-stained LLO were added at different MOIs and incubated for 4 h to determine number of cells with bacteria (a). BMDMs were analyzed for viability using flow cytometry at 4 and 12 h at a 50:1 MOI (b-c). Experiments were performed with three biological replicates (n=3) except for 25:1 and 100:1 MOI in uptake experiment (a) which were performed with one biological replicate (n=1) to correlate with live cell data trends. Data is mean ± SD; **p<0.01, ***p<0.001, ****p<0.0001, *****p<0.00001.

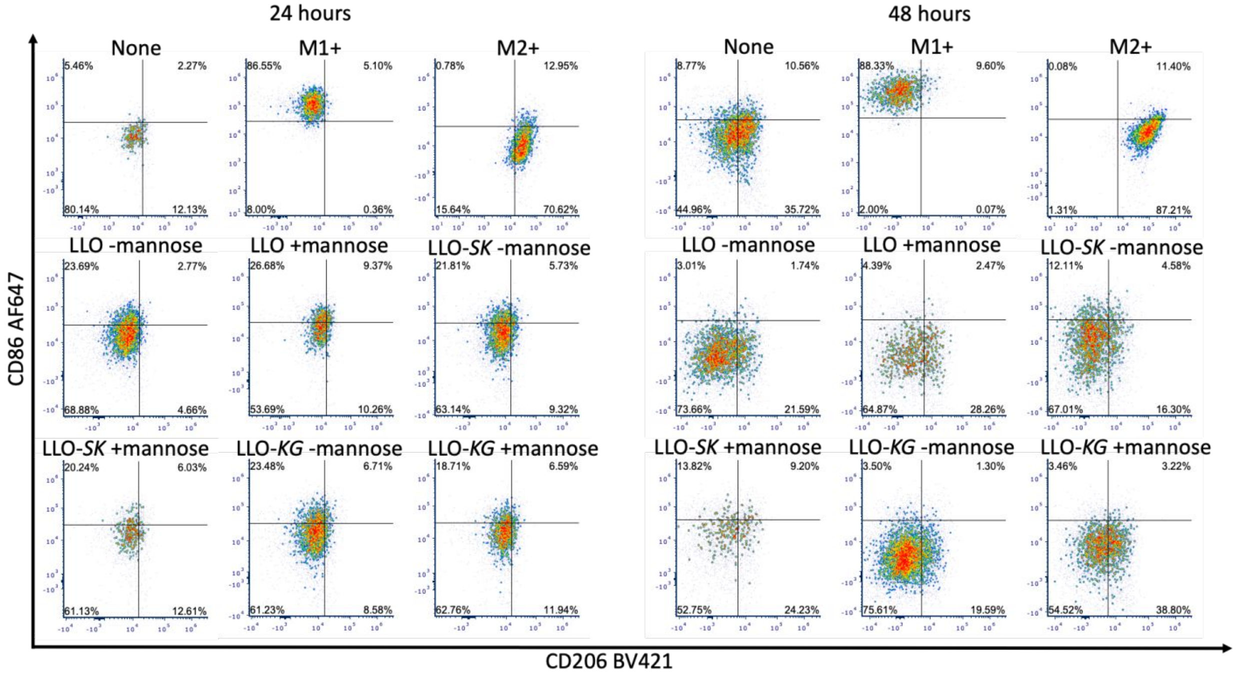

Supplementary Fig. S6. Changes in the levels of surface markers on BMDMs, by flow cytometry, after various treatments with *B. subtilis*

Flow cytometry dot plots of CD86 AF647 and CD206 BV421 surface markers after BMDMs were untreated (none), treated with LPS and IFN-γ (M1+), IL-4 and IL-13 (M2+), LLO strain with and without mannose (LLO -mannose, LLO +mannose), LLO-*SK* with and without mannose (LLO-*SK* -mannose, LLO-*SK* +mannose) and LLO-*KG* with and without mannose (LLO-*KG* -mannose, LLO-*KG* +mannose) at 24 and 48 h post-initial treatment. IPTG was added to all bacterial treatments. Percentages expressed in quadrants are from live, CD11b+/F4/80+ population.

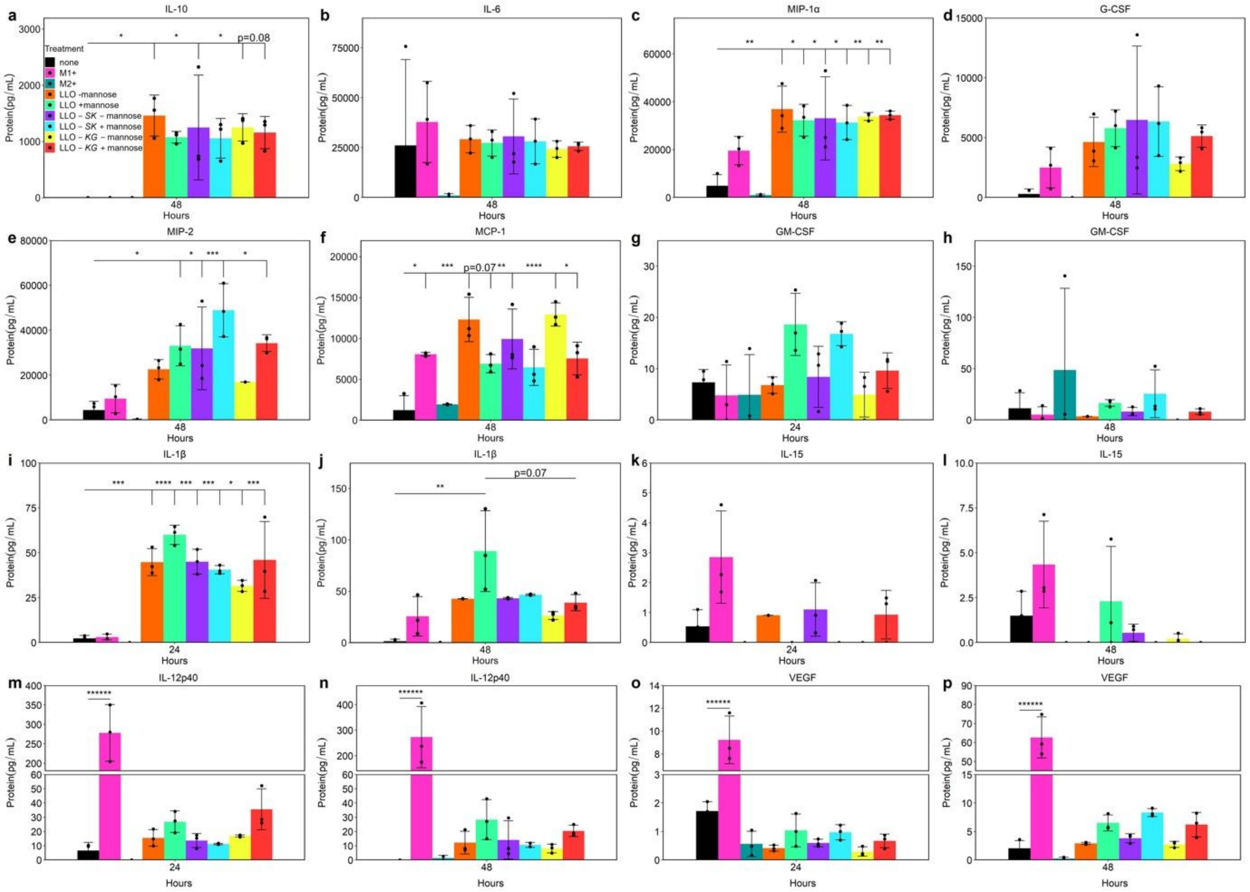

Supplementary Fig. S7. Additional profiling of BMDM cytokine and chemokine production after exposure to engineered *B. subtilis* LLO strains

Cytokine and chemokine protein concentrations were quantified after BMDMs cells were untreated (none), treated with LPS and IFN-γ (M1+), IL-4 and IL-13 (M2+), LLO strain with and without mannose (LLO -mannose, LLO +mannose), LLO-*SK* with and without mannose (LLO-*SK* -mannose, LLO-*SK* +mannose) and LLO-*KG* with and without mannose (LLO-*KG* -mannose, LLO-*KG* +mannose) at 24 h and 48 h post-initial treatment. IPTG was added to all bacterial treatments. IL-10 at 48 h (a), IL-6 at 48 h (b), MIP-1α at 48 h (c), G-CSF at 48 h (d), MIP-2 at 48 h (e), MCP-1 at 48 h (f), GM-CSF at 24 h (g), GM-CSF at 48 h (h), IL-1β at 24 h (i), IL-1β at 48 h (j), IL-15 at 24 h (k), IL-15 at 48 h (l), IL-12p40 at 24 h (m), IL-12p40 at 48 h (n), VEGF at 24 h (o) and VEGF at 48 h (p). Data is mean ± SD from n=3 biological replicates; *p<0.05, **p<0.01, ***p<0.001, ****p<0.0001, *****p<0.00001, ******p<0.000001.

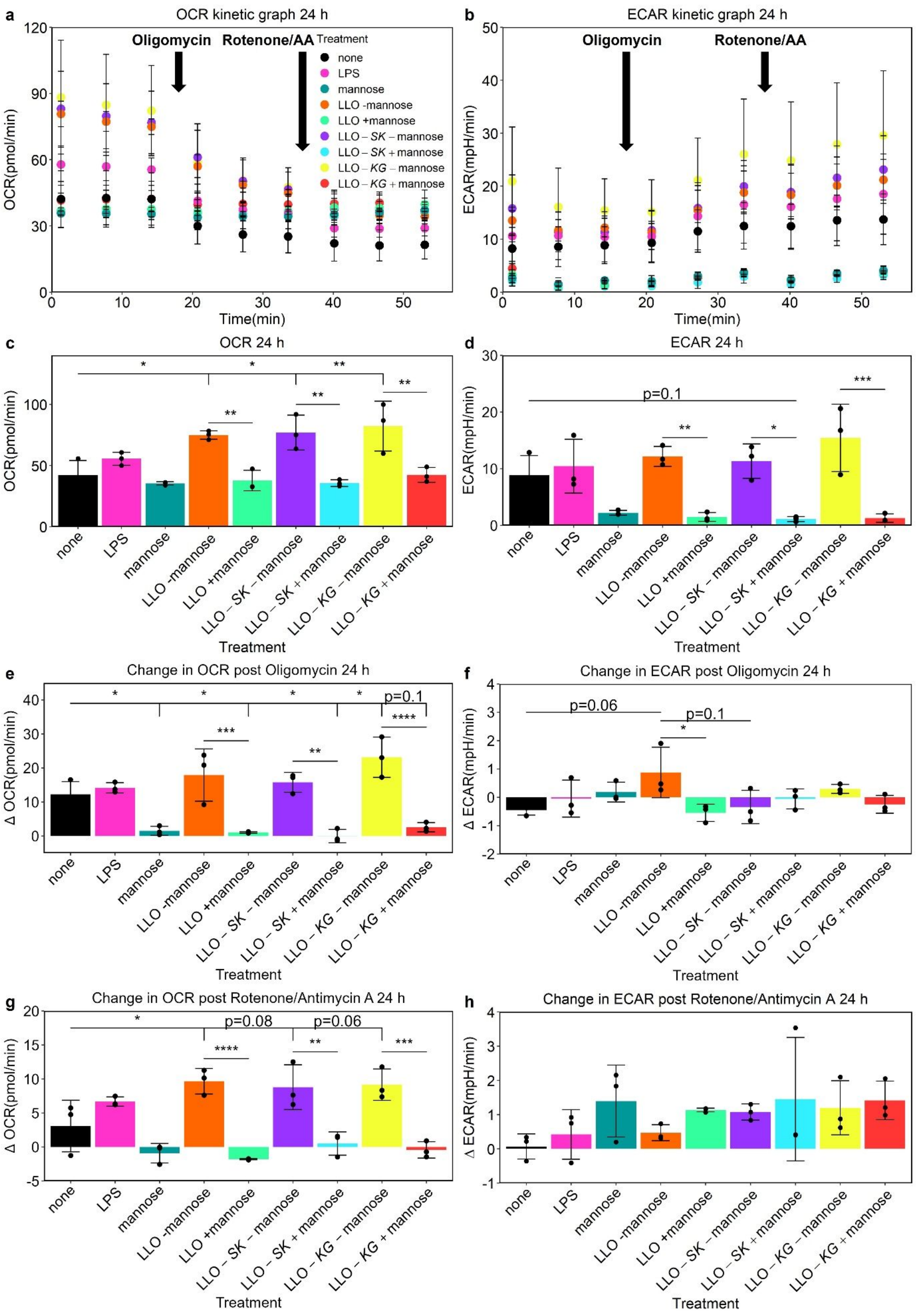
a
OCR kinetic graph 24 h
Oligomycin
Rotenone/AA
Treatment
none
LPS
mannose
LLO -mannose
LLO +mannose
LLO – SK – mannose
LLO – SK + mannose
LLO – KG – mannose
LLO – KG + mannose
OCR(pmol/min)
Time(min)
b
ECAR kinetic graph 24 h
Oligomycin
Rotenone/AA
ECAR(mpH/min)
Time(min)
c
OCR 24 h
OCR(pmol/min)
Treatment
d
ECAR 24 h
p=0.1
ECAR(mpH/min)
Treatment
e
Change in OCR post Oligomycin 24 h
p=0.1
Δ OCR(pmol/min)
Treatment
f
Change in ECAR post Oligomycin 24 h
p=0.06
p=0.1
Δ ECAR(mpH/min)
Treatment
g
Change in OCR post Rotenone/Antimycin A 24 h
p=0.08
p=0.06
Δ OCR(pmol/min)
Treatment
h
Change in ECAR post Rotenone/Antimycin A 24 h
Δ ECAR(mpH/min)
Treatment

Supplementary Fig. S8. Patterns of functional metabolism for BMDMs 24 h after exposure to engineered *B. subtilis* LLO variants

OCR and ECAR were measured before and after electron transport chain inhibitors, Oligomycin and Rotenone/antimycin A (AA), were added at points indicated on the kinetic plots (a, b). OCR and ECAR quantification at the third measurement before addition of inhibitors were plotted (c, d). Further analysis was performed to quantify changes in OCR (ΔOCR) and ECAR (ΔECAR) after inhibitors were added (e-h). BMDMs were untreated (none), treated with LPS, mannose, LLO strain with and without mannose (LLO -mannose, LLO +mannose), LLO-*SK* with and without mannose (LLO-*SK* -mannose, LLO-*SK* +mannose) and LLO-*KG* with and without mannose (LLO-*KG* -mannose, LLO-*KG* +mannose). IPTG was added to all bacterial treatments. Data is mean ± SD from n=3 biological replicates; *p<0.05, **p<0.01, ***p<0.001, ****p<0.0001.

Supplementary Table S1. Quantification of shifts in functional metabolism shifts between glycolysis and oxidative phosphorylation (as percent) and resulting differences in ATP production rates at 12 h

Quantification of shifts between glycolysis and oxidative phosphorylation (shown in percent) and resulting impact on ATP production (by two measures to create an index) when BMDMs were untreated (none), treated with LPS, mannose, LLO strain with and without mannose (LLO -mannose, LLO +mannose), LLO-*SK* with and without mannose (LLO-*SK* -mannose, LLO-*SK* +mannose) and LLO-*KG* with and without mannose (LLO-*KG* -mannose, LLO-*KG* +mannose) at 12 h post-addition of treatments.

	Basal Rates (Average) 12 h											
	glycoATP Production Rate(pmol/min)		mitoATP Production Rate (pmol/min)		Total ATP Production Rate(pmol/min)		XF ATP Rate Index		% Glycolysis		% Oxidative Phosphorylation	
Groups	Average	StDev	Average	StDev	Average	StDev	Average	StDev	Average	StDev	Average	StDev
Untreated (none)	7.4	3.2	28.3	11.2	35.8	13.7	4.1	1.6	20.9	6.5	79.1	6.5
LPS 100ng/ mL	38.7	7.7	67.3	18.0	106.0	25.7	1.7	0.1	36.8	1.5	63.2	1.5
Mannose	2.9	8.6	6.0	1.1	8.9	9.8	-2.596	3.810	-46.335	127.003	146.3	127.0
LLO	39.1	17.9	90.1	18.5	129.2	36.0	2.5	0.8	29.3	6.0	70.7	6.0
LLO +mannose	48.1	6.9	88.5	17.9	136.6	24.2	1.8	0.2	35.4	2.6	64.6	2.6
LLO-SK	31.9	15.5	87.2	27.2	119.1	42.2	2.9	0.5	26.1	3.7	73.9	3.7
LLO-SK +mannose	28.7	12.0	66.9	15.1	95.7	25.3	2.6	0.9	29.2	7.1	70.8	7.1
LLO-KG	60.3	17.1	125.3	34.0	185.6	51.0	2.1	0.1	32.5	1.0	67.5	1.0
LLO-KG +mannose	59.4	20.5	105.2	43.3	164.6	63.6	1.8	0.2	36.4	2.2	63.6	2.2

Supplementary Table S2. Quantification of shifts in functional metabolism between glycolysis and oxidative phosphorylation (as percent) and resulting differences in ATP production rates at 24 h

Quantification of shifts between glycolysis and oxidative phosphorylation (shown in percent) and resulting impact on ATP production (by two measures to create an index) when BMDMs were untreated (none), treated with LPS, mannose, LLO strain with and without mannose (LLO -mannose, LLO +mannose), LLO-*SK* with and without mannose (LLO-*SK* -mannose, LLO-*SK* +mannose) and LLO-*KG* with and without mannose (LLO-*KG* -mannose, LLO-*KG* +mannose) at 24 h post-addition of treatments.

	Basal Rates (Average) 24 h											
	glycoATP Production Rate(pmol/min)		mitoATP Production Rate (pmol/min)		Total ATP Production Rate(pmol/min)		XF ATP Rate Index		% Glycolysis		% Oxidative Phosphorylation	
Groups	Average	StDev	Average	StDev	Average	StDev	Average	StDev	Average	StDev	Average	StDev
Untreated	68.0	27.3	93.2	31.3	161.1	56.7	1.4	0.3	42.0	5.7	58.0	5.7
LPS 100ng/ mL	79.1	42.2	109.2	11.7	188.3	53.3	1.6	0.6	40.2	9.7	59.8	9.7
Mannose	20.1	4.1	8.3	7.4	28.5	11.3	0.38	0.27	74.2	14.4	25.8	14.4
LLO	86.3	15.0	165.0	13.0	251.4	26.5	1.9	0.2	34.2	2.6	65.8	2.6
LLO +mannose	13.3	6.8	5.9	1.2	19.2	6.9	0.52	0.28	67.3	11.6	32.7	11.6
LLO-SK	78.9	22.2	167.0	35.2	246.0	55.3	2.2	0.4	31.8	3.6	68.2	3.6
LLO-SK +mannose	9.4	3.9	5.3	6.1	14.7	10.0	0.44	0.45	74.3	23.8	25.7	23.8
LLO-KG	113.0	45.1	190.5	64.2	303.5	109.3	1.7	0.2	36.8	2.0	63.2	2.0
LLO-KG +mannose	10.5	6.0	14.6	8.9	25.1	14.8	1.4	0.2	42.0	4.3	58.0	4.3

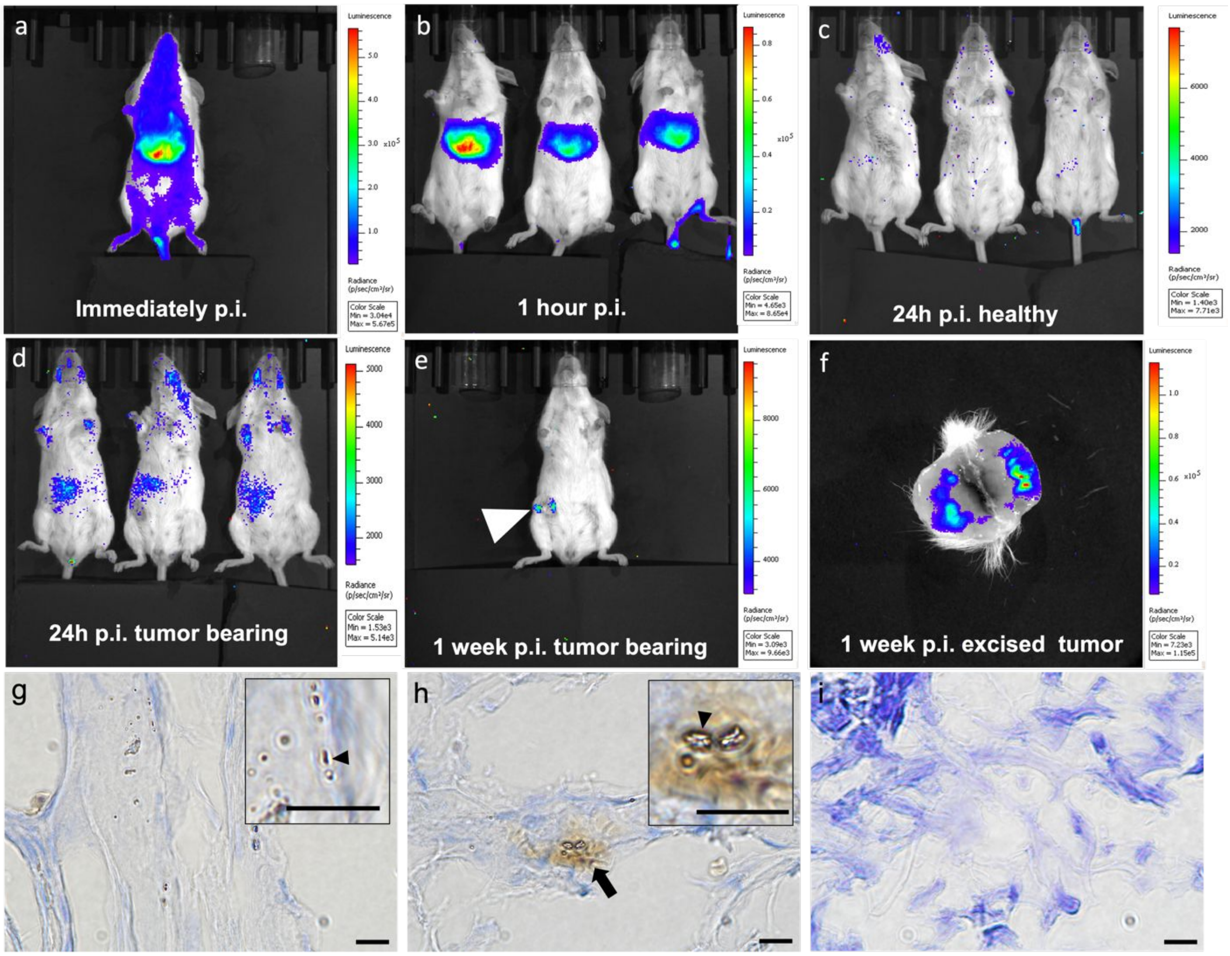

Supplementary Fig. S9*. In vivo* bioluminescence imaging of the LLO-*lux* strain in mice after intravenous injection into representative healthy and tumor bearing mice and end point tumor histology.

Representative healthy and 4T1 orthotopic tumor bearing mice were injected with 10^8 LLO-*lux* intravenously and followed using *in vivo* bioluminescence imaging (BLI) to monitor LLO-*lux* location and viability. Representative images are shown immediately after injection (a), then at 1 h post-injection (p.i.; b), 24 h p.i. (d) and 1-week p.i. (e, f). Healthy and tumor bearing mice showed the same trends immediately after injection and 1 h p.i. However, by 24 h, all bacterial signal is cleared from healthy mice (c). Bacteria were shown to persist throughout the tumor 1-week p.i. which was identified within the tumor in an intact animal (e) and in more detail, within an excised tumor which was cut in half (f). Immunohistochemistry identified rod-shaped bacteria (black arrowheads) in tumors which were injected intravenous (IV) with the bacteriotherapeutic. These were present both in CD11b- cells (g) and CD11b+ cells (h, brown). There were no bacteria observed in tumors of mice which did not receive bacteria injection (**i**). Scale bars = 20 µm.

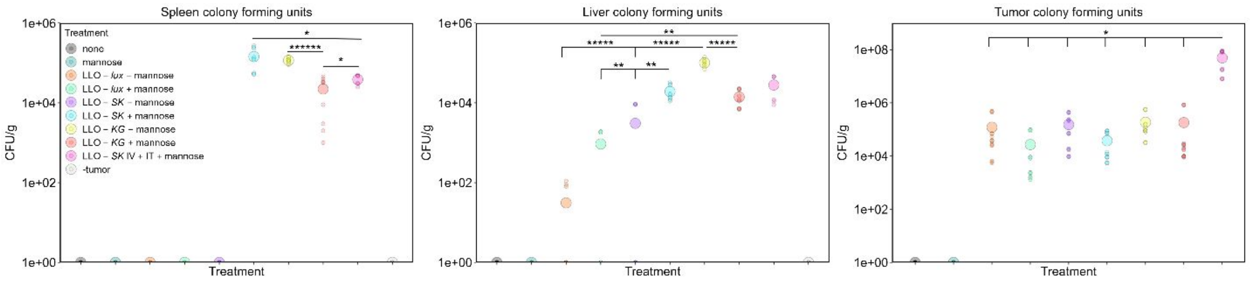

Supplementary Fig. S10. Colony forming units from tumors and relevant organs

Colony forming units (CFU) were calculated and normalized to mass (g) of spleens, livers and tumors. Groups were untreated (none), treated with mannose, LLO-*lux* strain injected IV with and without mannose (LLO-*lux* -mannose, LLO-*lux* +mannose), LLO-*SK* injected IV with and without mannose (LLO-*SK* -mannose, LLO-*SK* +mannose), LLO-*KG* injected IV with and without mannose (LLO-*KG* -mannose, LLO-*KG* +mannose), LLO-*SK* injected IV and IT with mannose (LLO-*SK* IV+IT +mannose) and no tumors (-tumor). IPTG was added to all bacterial treatments. Data is a scatter plot of all individual values with large point representing the mean from n=3 spleens/livers and n=5 tumors (3 halves and 2 whole) that were plated in triplicate from each treatment.

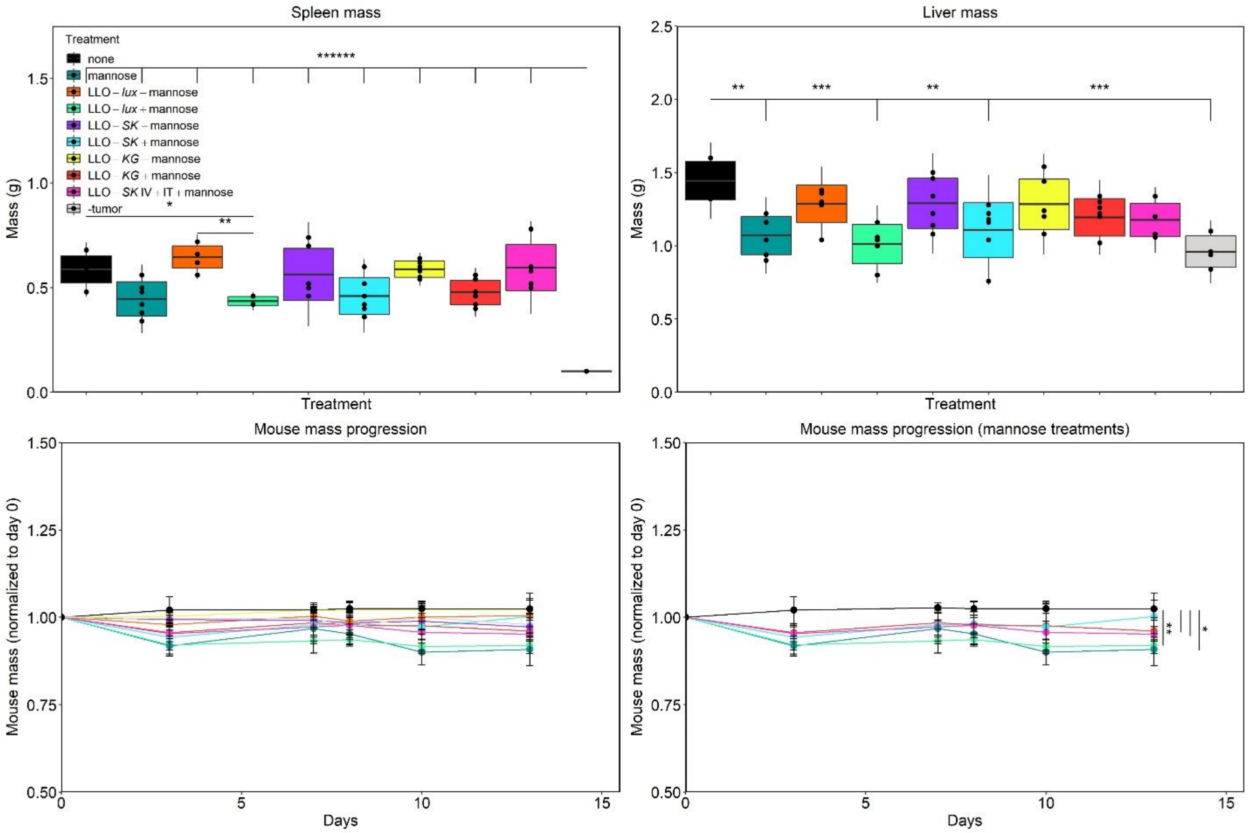

Supplementary Fig. S11. Changes in body weight (mass progression) of mice and final mass of organs found to contain bacteria

The mass of spleens and liver (top panels) and mice (lower panels) after the following treatments: untreated (none), treated with mannose, LLO-*lux* strain injected IV with and without mannose (LLO-*lux* -mannose, LLO-*lux* +mannose), LLO-*SK* injected IV with and without mannose (LLO-*SK* -mannose, LLO-*SK* +mannose), LLO-*KG* injected IV with and without mannose (LLO-*KG* -mannose, LLO-*KG* +mannose), LLO-*SK* injected IV and IT with mannose (LLO-*SK* IV+IT +mannose) and healthy mice injected with LLO-*lux* (-tumor). IPTG was added to all bacterial treatments. Data is mean ± SD for box and two times ± SD for whiskers from n=6 mice (tumor bearing) and n=4 mice (-tumor) for upper panels and mean ± SD from same mice for lower panels.

Supplementary Table S3. Summary of liver histopathology

The livers of tumor-bearing mice in five treatment groups [1 (untreated), 2 (mannose-treated), 4 (LLO +mannose), 6 (LLO-*SK* +mannose) and 8 (LLO-*KG* +mannose)] were excised, fixed, sectioned, processed for H&E staining and examined by a pathologist. Treatment conditions (+: present, -: not present) are indicated in table including the mice which received bacteria and/or Mannose/IPTG. Histopathology and observations are described in the table. Giemsa staining was performed in an attempt to stain bacteria; none were found in any group (-).

Group	Primary tumor	Bacteria	Mannose/IPTG	Histopathology findings	Bacteria found
G1	+	-	-	Multifocal perivascular and random mononuclear and neutrophilic cell infiltrates	-
G2	+	-	+	Multifocal perivascular and random mononuclear and neutrophilic cell infiltrates	-
G4	+	+ (LLO)	+	Multifocal perivascular and random mononuclear and neutrophilic cell infiltrates	-
G6	+	+ (SK)	+	Multifocal perivascular and random mononuclear and neutrophilic cell infiltrates	-
G8	+	+ (KG)	+	Multifocal perivascular and random mononuclear and neutrophilic cell infiltrates	-

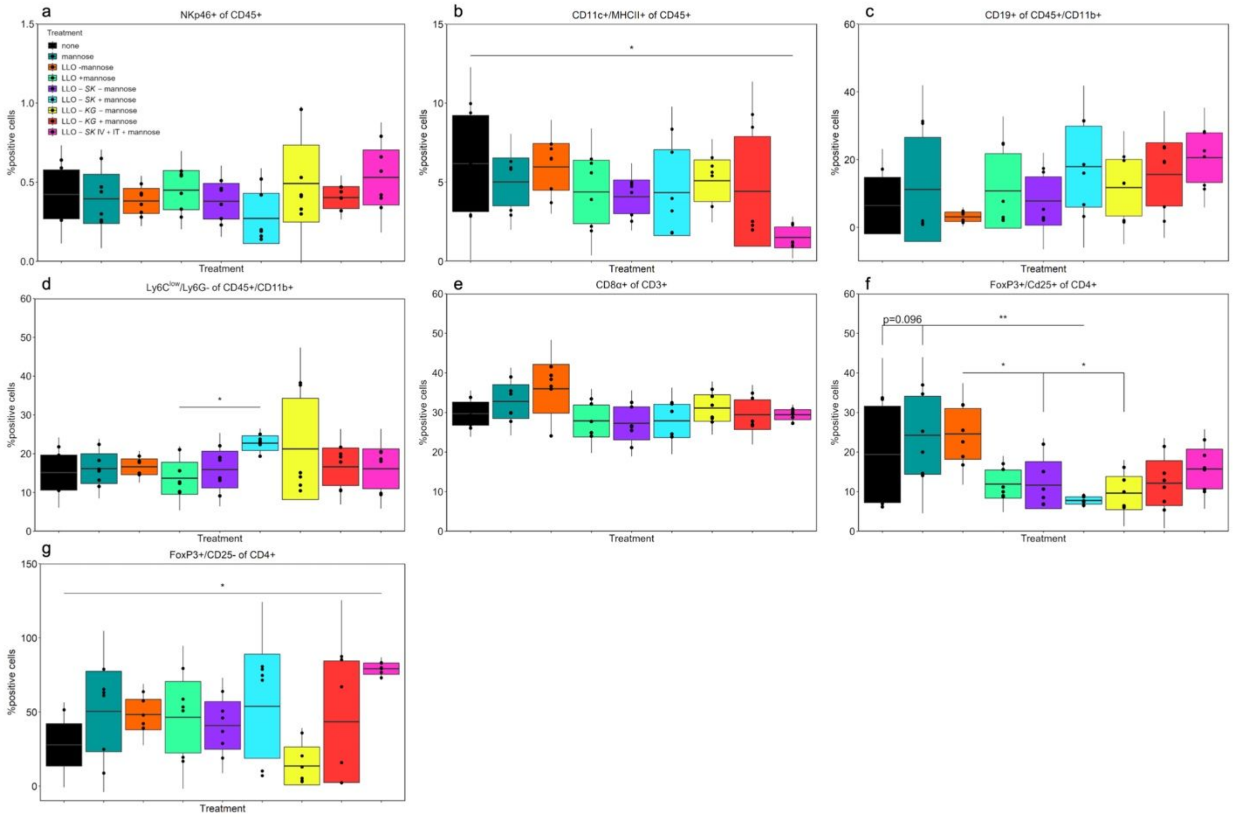

Supplementary Fig. S12. Immunophenotyping of 4T1 tumors after treatment

Immune cell populations were analyzed using flow cytometry, from digested tumors from all treatment groups: untreated (none), treated with mannose, LLO-*lux* strain injected IV with and without mannose (LLO-*lux* -mannose, LLO-*lux* +mannose), LLO-*SK* injected IV with and without mannose (LLO-*SK* -mannose, LLO-*SK* +mannose), LLO-*KG* injected IV with and without mannose (LLO-*KG* -mannose, LLO-*KG* +mannose) and LLO-*SK* injected IV and IT with mannose (LLO-*SK* IV+IT +mannose). IPTG was added to all bacterial treatments. Populations analyzed are NK cells (NKp46$^+$ of CD45$^+$, a), dendritic cells (CD11c$^+$/MHCII$^+$ of CD45$^+$, b), B cells (CD19$^+$ of CD45$^+$/CD11b$^+$, c), tumor associated macrophages (TAMs; Ly6C^{low}/Ly6G$^-$ of CD45$^+$/CD11b$^+$, d), cytotoxic T cells (CD8α^+ of CD3$^+$, e), T regulatory cells (Treg; FoxP3$^+$/CD25$^+$ of CD4$^+$, f) and an alternative Treg subset (FoxP3$^+$/CD25$^-$ of CD4$^+$, g). Data is mean ± SD for box and two times ± SD for whiskers from n=3 tumors in technical duplicates for each treatment; *p<0.05, **p<0.01.

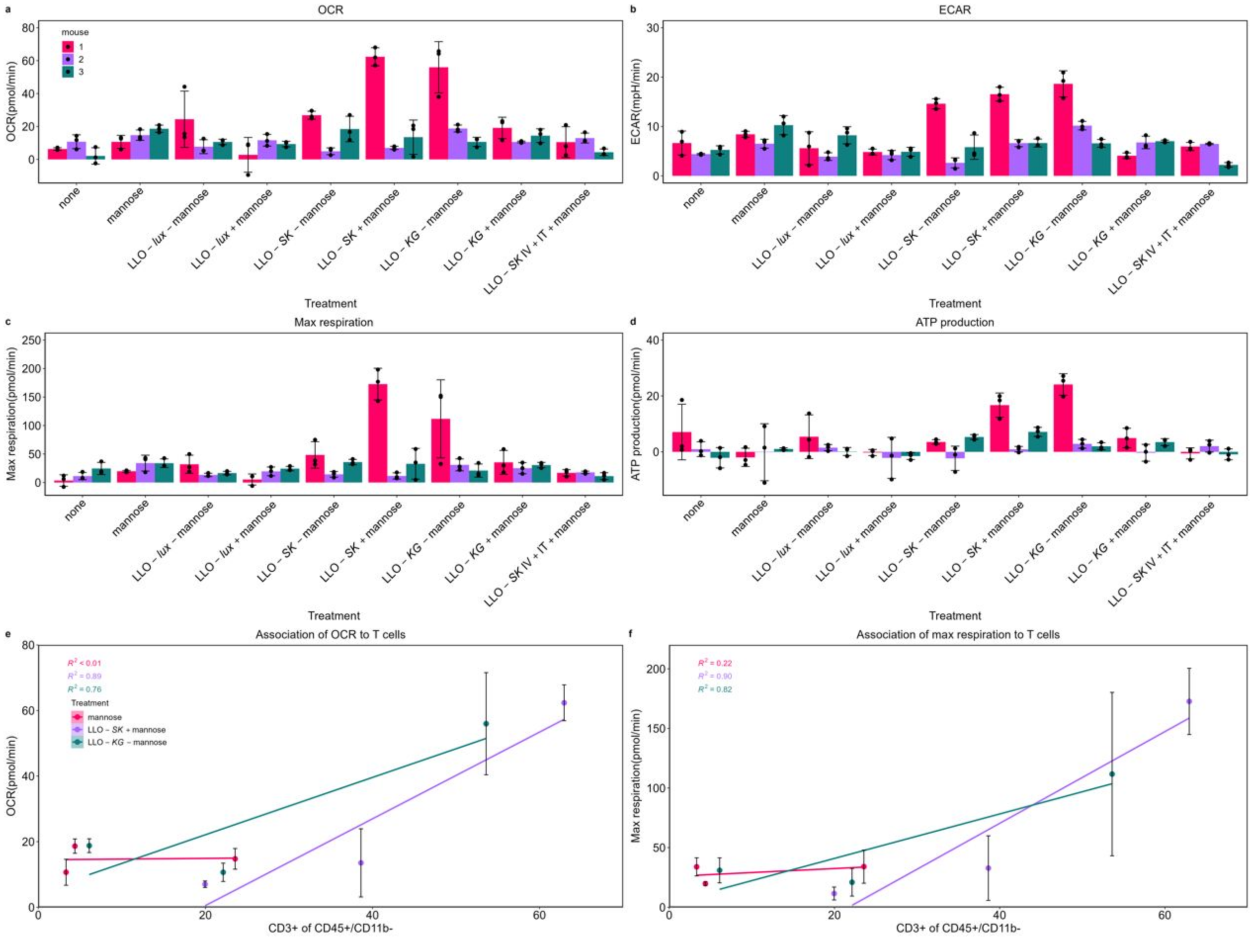

Supplementary Fig. S13. Functional metabolism of cells in the tumor microenvironment after treatment

OCR, ECAR, max respiration and ATP production were measured from cells released from resected tumors from treated mice (n=3 mice, n=3 technical replicates). Individual measurements from the tumors from each mouse were plotted and grouped into treatment schemes (a-d). Example associations between OCR (e) or Max respiration (f) measurements and CD3+ T cells (taken from the same tumors, split in half for each analysis) are plotted with the two treatment groups, with the highest association, and one with a lower association shown. R^2 values are noted for each treatment group. Data is mean ± SD.
